# The global impact of household contact management for children on multidrug-resistant and rifampicin-resistant tuberculosis cases, deaths, and health-system costs in 2019: a modelling study

**DOI:** 10.1016/S2214-109X(22)00113-9

**Published:** 2022-05-18

**Authors:** Peter J Dodd, Nyashadzaishe Mafirakureva, James A Seddon, Christopher F McQuaid

**Affiliations:** a School of Health and Related Research, University of Sheffield, Sheffield, UK; b Desmond Tutu TB Centre, Department of Paediatrics and Child Health, Stellenbosch University, Cape Town, South Africa; c Department of Infectious Diseases, Imperial College London, London, UK; d TB Modelling Group, TB Centre and Centre for Mathematical Modelling of Infectious Diseases, Department of Infectious Disease Epidemiology, Faculty of Epidemiology and Population Health, London School of Hygiene & Tropical Medicine, London, UK

## Abstract

**Background:**

Estimates suggest that at least 30 000 children develop multidrug-resistant or rifampicin-resistant tuberculosis each year. Despite household contact management (HCM) being widely recommended, it is rarely done.

**Methods:**

We used mathematical modelling to evaluate the potential country-level and global effects and cost-effectiveness of multidrug-resistant or rifampicin-resistant tuberculosis HCM for children younger than 15 years who are living with a person with newly diagnosed multidrug-resistant or rifampicin-resistant tuberculosis. We compared a baseline of no HCM with several HCM strategies and tuberculosis preventive therapy regimens, calculating the effect on multidrug-resistant or rifampicin-resistant tuberculosis cases, deaths, and health-system costs. All HCM strategies involved the screening of children for prevalent tuberculosis disease but with tuberculosis preventive therapy either not given or targeted dependent on age, HIV status, and result of tuberculin skin test. We evaluated the use of fluoroquinolones (ie, levofloxacin and moxifloxacin), delamanid, and bedaquiline as tuberculosis preventive therapy.

**Findings:**

Compared with a baseline without HCM, HCM for all adults diagnosed with multidrug-resistant or rifampicin-resistant tuberculosis in 2019 would have entailed screening 227 000 children (95% uncertainty interval [UI]: 205 000–252 000) younger than 15 years globally, and averted 2350 tuberculosis deaths (1940–2790), costing an additional US$63 million (74–95 million). If all the children within the household who had been in contact with the person with multidrug-resistant or rifampicin-resistant tuberculosis received tuberculosis preventive therapy with levofloxacin, 5620 incident tuberculosis cases (95% UI 4540–6890) and an additional 1240 deaths (970–1540) would have been prevented. Incremental cost-effectiveness ratios were lower than half of per-capita gross domestic product for most interventions in most countries. Targeting only children younger than 5 years and those living with HIV reduced the number of incident cases and deaths averted, but improved cost-effectiveness. Tuberculosis preventive therapy with delamanid increased the effect, in terms of reduced incidence and mortality, compared with levofloxacin.

**Interpretation:**

HCM for patients with multidrug-resistant or rifampicin-resistant tuberculosis is cost-effective in most settings and could avert a substantial proportion of multidrug-resistant or rifampicin-resistant tuberculosis cases and deaths in children globally.

**Funding:**

UK Medical Research Council.

## Introduction

Estimates by WHO suggest that more than 1 million children become ill with tuberculosis each year, yet only about half of these are diagnosed and treated.^1^ Nearly a quarter of children with tuberculosis die; almost all of these are undiagnosed, resulting in tuberculosis being a leading cause of child mortality.^2^ Modelling estimates suggest that approximately 30 000 children develop multidrug-resistant tuberculosis (defined as disease caused by *Mycobacterium tuberculosis* that is resistant to at least isoniazid and rifampicin) each year.^3^, ^4^ If appropriately treated, the outcomes of multidrug-resistant tuberculosis are good (>90% treatment success).^5^ However, only 5600 children were estimated to have been identified with multidrug-resistant or rifampicin-resistant tuberculosis in 2019, corresponding to a case detection ratio of at best 19%.^1^ In comparison, 39% of adult multidrug-resistant or rifampicin-resistant cases are estimated to be identified and treated.^1^ Although estimates of death from multidrug-resistant or rifampicin-resistant tuberculosis in children are not available, this failure to diagnose and treat children with multidrug-resistant or rifampicin-resistant tuberculosis probably leads to a substantial number of child deaths. Data from before the chemotherapy era suggest that mortality in children younger than 5 years with untreated tuberculosis is more than 40%.^6^ Estimates also suggest that children younger than 15 years with tuberculosis infection are 10 times more likely to have a multidrug-resistant or rifampicin-resistant tuberculosis infection than are infected adults.^7^ 600 000 children were estimated to be infected with latent multidrug-resistant or rifampicin-resistant tuberculosis infection in 2013 and 2014,^7^ and up to 19% of those younger than 5 years and 10% of those aged 5–14 years will have progressed to tuberculosis disease.^8^ Children, therefore, continue to represent a key marginalised group who are susceptible to multidrug-resistant or rifampicin-resistant tuberculosis.

**Research in context**

**Evidence before this study**

We searched PubMed with the terms (“TB” OR “tubercul*”) AND (“resist*” OR “multidrug*” OR “MDR*”) AND (“household” OR “prevent*”) AND “contact*” AND (“model*” OR “cost-effect*”) for articles published from database inception to Nov 1, 2021, in English. We found 69 studies, from which we identified two studies that had estimated the cost-effectiveness of preventive therapy alone for drug-resistant tuberculosis. No studies considered the effect or cost-effectiveness of household contact management. Marks and colleagues conducted a systematic review and meta-analysis of the efficacy and cost-effectiveness of preventive therapy for multidrug-resistant tuberculosis, and did a simple societal perspective cost-effectiveness analysis of the five regimens that were identified (disregarding the effect of case finding), finding that most were cost saving. Meanwhile, Fox and colleagues calculated the cost-effectiveness of fluoroquinolone preventive therapy in the USA and reported that it was cost saving, even if effectiveness was low. Shah and colleagues conducted a systematic review and meta-analysis of the prevalence of tuberculosis in contacts of patients with multidrug-resistant tuberculosis and reported that nearly 8% of household contacts had co-prevalent tuberculosis disease. Several studies were identified that compared the infectiousness and virulence of drug-susceptible versus drug-resistant *Mycobacterium tuberculosis*. This topic has been systematically reviewed by Kodama and colleagues, who found no evidence that drug-resistant tuberculosis resulted in fewer cases of tuberculosis disease in household contacts than did drug-susceptible tuberculosis. Huang and colleagues evaluated the use of isoniazid preventive therapy in contacts of patients with multidrug tuberculosis and reported that its use lowered the incidence of tuberculosis disease. Seddon and colleagues described a trial protocol for the use of levofloxacin preventive therapy in child contacts of patients with multidrug-resistant tuberculosis.

**Added value of this study**

Our study is the first to evaluate the cost-effectiveness of household contact management for children living with patients with multidrug-resistant or rifampicin-resistant tuberculosis, including case finding for prevalent tuberculosis disease and considering 213 countries with varying rates of fluoroquinolone resistance. We compare different preventive therapy regimens and recipients, providing country-level estimates of health benefits, costs to health systems, and incremental cost-effectiveness ratios.

**Implications of all the available evidence**

Household contact management for patients with multidrug-resistant or rifampicin-resistant tuberculosis could have averted as many as 3950 child tuberculosis deaths in 2019, at a global aggregate cost-effectiveness of US$1208 per disability-adjusted life-year averted. Household contact tracing for multidrug-resistant or rifampicin-resistant tuberculosis is likely to have substantial health benefits and be cost-effective in many settings, with adoption and approach informed by forthcoming trial results and local implementation research.

Although only 10–30% of children with tuberculosis are thought to acquire infection in their household,^9^ household contact management (HCM) is a highly efficient strategy for the identification of children with tuberculosis infection and disease, with nearly 8% of household contacts of drug-resistant tuberculosis patients having co-prevalent tuberculosis at the time of evaluation, and nearly half of contacts having evidence of tuberculosis infection.^10^ HCM seeks to identify and treat co-prevalent tuberculosis disease and, if disease is excluded, offer tuberculosis preventive therapy to contacts who are deemed at high risk for progression to incident tuberculosis.^11^ HCM following identification of individuals with both rifampicin-susceptible and multidrug-resistant or rifampicin-resistant tuberculosis disease is recommended by WHO and most national tuberculosis programmes,^12^ often with high priority when the index patient has multidrug-resistant or rifampicin-resistant tuberculosis. Children younger than 5 years, living with HIV, or with positive tuberculin skin test (TST) are at particularly high risk of incident tuberculosis.^8^ In practice though, systematic HCM coverage is low in most low-income and middle-income countries.^1^

For close contacts of patients with rifampicin-susceptible tuberculosis, guidelines recommend tuberculosis preventive therapy with isoniazid, a rifamycin, or a combination of the two.^12^ However, all WHO-recommended regimens contain drugs to which multidrug-resistant or rifampicin-resistant organisms are resistant. Existing WHO policy recommendations for patients with multidrug-resistant or rifampicin-resistant tuberculosis infection suggest only close monitoring and possible tuberculosis preventive therapy on the basis of clinical judgment for those at high risk of disease progression.^12^ Scarce evidence that is limited by its observational nature suggests that tuberculosis preventive therapy directed at the drug susceptibility test pattern of the strain from the index patient is likely to be safe and results in low risk of incident disease.^13^, ^14^, ^15^, ^16^ Three separate randomised controlled trials of tuberculosis preventive therapy for household contacts of index patients with multidrug-resistant or rifampicin-resistant tuberculosis are ongoing: two evaluating levofloxacin^17^, ^18^ and one evaluating delamanid (NCT03568383). However, even if effective in preventing incident multidrug-resistant or rifampicin-resistant tuberculosis, it is unclear what the effect of these potential preventive therapies would be if scaled up. Although resistance to delamanid and bedaquiline is low in most parts of the world, around 20% of patients with multidrug-resistant or rifampicin-resistant tuberculosis also have resistance to fluoroquinolones.^1^ Improved understanding of the country-level impact and cost-effectiveness of the different components of HCM (eg, diagnosis of co-prevalent disease *vs* the provision of multidrug-resistant or rifampicin-resistant tuberculosis preventive therapy), and the different regimens available, would help to inform policy makers.

Motivated by the low uptake of HCM for multidrug-resistant or rifampicin-resistant tuberculosis, impending tuberculosis preventive therapy study results, and the need to provide evidence that includes costs when considering multidrug-resistant or rifampicin-resistant tuberculosis, we aimed to evaluate the potential effect and cost-effectiveness of universal multidrug-resistant or rifampicin-resistant tuberculosis HCM for children compared with the current standard of care, where HCM rarely occurs. We report global and country-level results for strategies, including different multidrug-resistant or rifampicin-resistant tuberculosis preventive therapy recipient groups and different regimens.

## Methods

**Modelling approach**

**Figure 1:**
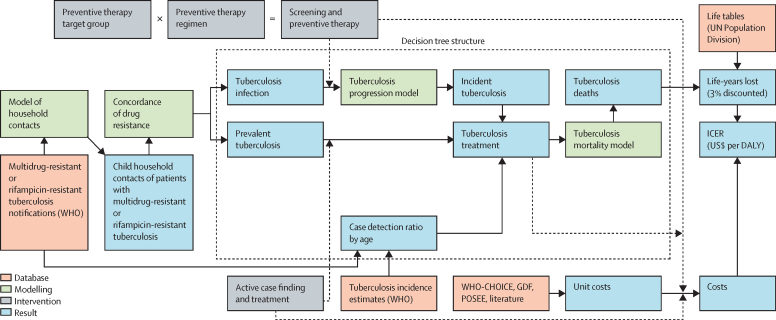
Modelling logic overview The dotted box shows elements of the overall model that are assessed using the decision tree model. Solid arrows indicate flow of data. Dashed arrows indicate changes under the intervention. US$ are 2020 US$. WHO-CHOICE=WHO-Choosing Interventions that are Cost-Effective. GDF=Global Drug Facility. POSEE=Paediatric Operational Sustainability Expertise Exchange. DALY=disability-adjusted life-year. ICER=incremental cost-effectiveness ratio.

We adapted a previous model of tuberculosis HCM^11^ to include different antituberculosis drug-resistance types and incorporate updated evidence and costs (figure 1). We focused on index patients with multidrug-resistant or rifampicin-resistant tuberculosis in 213 countries in 2019, on the basis of notification data reported to WHO. We assumed that the age and sex pattern of these patients was the same as other patients with tuberculosis, and used these data with estimates of the number of household contacts aged 0–4 years and 5–14 years for each sex and age group from the regression model developed by Dodd and colleagues.^11^

For this analysis, we based co-prevalence on a systematic review and meta-analysis specific to drug-resistant tuberculosis.^10^ We also updated the risks of progression to incident tuberculosis for contacts using a large individual-patient meta-analysis.^8^ Our model of latent tuberculosis infection risk by age and country was unchanged.

A proportion of co-prevalent or incident tuberculosis among household contacts was assumed to be rifampicin susceptible (ie, non-concordant), unvarying by country, and 83% concordant (based on data from Chiang and colleagues).^19^ The proportion of household contacts with multidrug-resistant or rifampicin-resistant tuberculosis who also had fluoroquinolone resistance in each country was based on WHO data, using a Bayesian scheme to estimate the proportion of rifampicin-resistant tuberculosis with fluoroquinolone resistance in each country^3^ (appendix pp 3–5). The probability that tuberculosis in child contacts was detected and was treated without HCM was based on the country-specific and age-specific WHO case detection ratio but inflated upwards by a random value between 1 and 2, truncating at 1,^11^ because notified index patients' contacts might have above-average likelihood of detection. We used the WHO-estimated proportion of patients with multidrug-resistant or rifampicin-resistant tuberculosis receiving multidrug-resistant or rifampicin-resistant treatment in each country to model the probability that detected patients with multidrug-resistant or rifampicin-resistant tuberculosis would receive appropriate treatment.^1^ We did not model secondary transmission by children, whom we considered non-infectious.

Outcomes of tuberculosis disease were based on meta-analyses specific to first-line or second-line treatment and stratified by HIV and antiretroviral therapy status.^5^, ^6^ Tuberculosis preventive therapy with a fluoroquinolone was assumed to be as effective as tuberculosis preventive therapy for patients with rifampicin-susceptible tuberculosis when treating patients with fluoroquinolone-susceptible multidrug-resistant or rifampicin-resistant tuberculosis,^8^ stratified by HIV and TST positivity. However, tuberculosis preventive therapy with a fluoroquinolone was assumed to have no efficacy against fluoroquinolone-resistant organisms. Bedaquiline and delamanid were assumed to be as effective as tuberculosis preventive therapy for rifampicin-susceptible tuberculosis against any tuberculosis strain.

All model parameters were considered to be uncertain and described by probability distributions; 1000 samples of all inputs were used for probabilistic uncertainty analysis, with means and uncertainty intervals (as 95% quantiles) reported. Calculations were performed with a decision tree framework in R. Additional detail is provided in the appendix (pp 2–13), which also reports results for a model without drug-resistance for comparison, and a sensitivity analysis assuming that not all index patients with multidrug-resistant or rifampicin-resistant tuberculosis have pulmonary disease. All analysis code and data are available on GitHub.

### Interventions considered

In our standard of care (baseline) scenario, we assumed that no HCM was conducted and no tuberculosis preventive therapy was offered. Any contacts with co-prevalent or incident tuberculosis were assumed to be detected, tested for drug resistance, and treated (assuming the usual country-specific case detection rate for household contacts).

Interventions considered either HCM without tuberculosis preventive therapy, or three different groups of tuberculosis preventive therapy recipients: children younger than 5 years or younger than 15 years living with HIV, children younger than 5 years or those younger than 15 years who were either living with HIV or had a positive TST, or all children younger than 15 years. For interventions with tuberculosis preventive therapy, we considered four different regimens: levofloxacin or moxifloxacin (presumed to have the same efficacy but different costs) or bedaquiline or delamanid (similarly assumed to have the same efficacy but different costs). There was therefore a total of 13 interventions to compare with standard of care. In all 13 scenarios, household contacts were assumed to be screened for tuberculosis disease, with all co-prevalent disease treated with the same regimen as the index patient. Full coverage (ie, HCM applied for all detected rifampicin-resistant and multidrug-resistant index cases) was used to allow transparent comparisons between interventions. In each of the scenarios in which tuberculosis preventive therapy was used, we assumed that the same drug was given to all recipients, with regimens having different efficacy against rifampicin-susceptible tuberculosis, multidrug-resistant or rifampicin-resistant tuberculosis, and fluoroquinolone-resistant multidrug-resistant or rifampicin-resistant tuberculosis. The appendix (pp 14–15) shows a summary of intervention activities.

### Health economics

For each intervention scenario, we calculated the resource requirements, health outcomes (including incident tuberculosis by drug-resistance status, and incident and co-prevalent deaths due to tuberculosis), health system costs, discounted life-years lost, and cost-effectiveness compared with the baseline scenario from a health system perspective.

For each intervention, we included the cost of a household visit and screening and assumed that some children would screen positive and require an outpatient visit with chest x-ray and GeneXpert MTB/RIF testing. Interventions with tuberculosis preventive therapy included costs of TSTs (if applicable), HIV tests, and then costs of regimen drugs, follow-up, monitoring, and management of adverse events. Resources used in antituberculosis treatment included contributions from HIV testing, laboratory monitoring, outpatient and any inpatient care, national tuberculosis programme costs, and the costs of drugs for each regimen in each age group (appendix pp 13–19).

Regression analysis was used to generate national tuberculosis programme and inpatient and outpatient costs for countries with missing data. All costs were adjusted for inflation and reported in 2020 US dollars. We assumed that costs accrue in the present with no discounting applied. Additional details can be found in the appendix (pp 19–21).

We calculated disability-adjusted life-years (DALYs) neglecting morbidity for each country, discounted at 3% per year on the basis of UN estimates over a lifetime horizon, and assuming uniform age distribution within age categories of 0–4 years and 5–14 years. Sensitivity analyses used 1% and 5% per year. Discounted life-years lost were then used to compute incremental cost-effectiveness ratios (ICERs) for each country in US dollars per DALY averted.

For each intervention, we report absolute and incremental numbers screened (compared with no intervention) and receiving tuberculosis preventive therapy or first-line or second-line antituberculosis treatment globally; the absolute and incremental tuberculosis incidence (by drug-susceptibility status) and deaths (by whether deaths were among incident or co-prevalent tuberculosis); and the absolute and incremental costs, life-years lost, and ICERs. We report the number of household visits or courses of tuberculosis preventive therapy needed to prevent one death or episode of incident tuberculosis, and the variation by prevalence of fluoroquinolone resistance. Finally, we report the country-level ICERs for each intervention and the probability of being cost-effective at different thresholds, and we provide contextual information on threshold choice.

### Role of the funding source

The funder of the study had no role in study design, data collection, data analysis, data interpretation, or writing of the report.

## Results

If HCM had been conducted for all adults diagnosed with multidrug-resistant or rifampicin-resistant tuberculosis in 2019, we projected that the number of households contacts younger than 15 years globally would have been 227 000 (95% uncertainty interval [UI] 205 000–252 000). Providing tuberculosis preventive therapy to children younger than 5 years or younger than 15 years and living with HIV would have required 71 200 courses (95% UI 63 400–79 000), whereas including children younger than 15 years with positive TST would have required 144 000 courses (129 000–160 000), and providing tuberculosis preventive therapy for all children younger than 15 years would have required 209 000 courses (189 000–232 000; table 1). In the absence of HCM, 12 700 contacts (95% UI 10 100–15 600) younger than 15 years would have received treatment for rifampicin-susceptible tuberculosis and 5170 contacts (3570–7240) younger than 15 years would have received treatment for multidrug-resistant or rifampicin-resistant tuberculosis. HCM without the provision of tuberculosis preventive therapy would have resulted in 7930 contacts (95% UI 6310–9890) younger than 15 years receiving treatment for rifampicin-susceptible tuberculosis and 16 700 contacts (14 500–19 100) younger than 15 years receiving treatment for multidrug-resistant or rifampicin-resistant tuberculosis due to improved detection and identification as multidrug-resistant or rifampicin-resistant tuberculosis.

**Table 1:** Total and incremental resources, incidence, and deaths for global household contact management interventions for multidrug-resistant and rifampicin-resistant tuberculosis in children younger than 15 years in 2019

	**No intervention**	**HCM only**	**HCM and TPT for all children aged <5 years or <15 years living with HIV**		**HCM and TPT for all children aged <5 years or <15 years living with HIV or with positive tuberculin skin test**		**HCM and TPT for all children aged <15 years**	
			Fluoroquinolone*	Bedaquiline or delamanid	Fluoroquinolone*	Bedaquiline or delamanid	Fluoroquinolone*	Bedaquiline or delamanid
**Total resources**								
Household contacts screened	0	227 000 (205 000 to 252 000)	227 000 (205 000 to 252 000)	227 000 (205 000 to 252 000)	227 000 (205 000 to 252 000)	227 000 (205 000 to 252 000)	227 000 (205 000 to 252 000)	227 000 (205 000 to 252 000)
TPT courses	0	0	71 200 (63 400 to 79 100)	71 200 (63 400 to 79 100)	144 000 (129 000 to 160 000)	144 000 (129 000 to 160 000)	209 000 (189 000 to 232 000)	209 000 (189 000 to 232 000)
Rifampicin-susceptible tuberculosis treatments	12 700 (10 100 to 15 600)	7930 (6310 to 9890)	7010 (5480 to 8870)	6830 (5310 to 8720)	5770 (4570 to 7160)	5340 (4270 to 6570)	5410 (4290 to 6740)	4910 (3930 to 5970)
Multidrug-resistant or rifampicin-resistant tuberculosis treatments	5170 (3570 to 7240)	16 700 (14 500 to 19 100)	16 400 (14 300 to 18 800)	16 300 (14 200 to 18 700)	16 000 (14 100 to 18 200)	15 700 (13 700 to 17 800)	15 900 (14 000 to 18 100)	15 500 (13 500 to 17 600)
**Incremental resources**								
Household contacts screened	Reference	227 000 (205 000 to 252 000)	227 000 (205 000 to 252 000)	227 000 (205 000 to 252 000)	227 000 (205 000 to 252 000)	227 000 (205 000 to 252 000)	227 000 (205 000 to 252 000)	227 000 (205 000 to 252 000)
TPT courses	Reference	0	71 200 (63 400 to 79 100)	71 200 (63 400 to 79 100)	144 000 (129 000 to 160 000)	144 000 (129 000 to 160 000)	209 000 (189 000 to 232 000)	209 000 (189 000 to 232 000)
Rifampicin-susceptible tuberculosis treatments	Reference	−4770 (−6980 to −2900)	−5690 (−8010 to −3800)	−5870 (−8220 to −3980)	−6930 (−9390 to −4940)	−7360 (−9820 to −5290)	−7290 (−9780 to −5250)	−7800 (−10 300 to −5670)
Multidrug-resistant or rifampicin-resistant tuberculosis treatments	Reference	11 600 (9360 to 13 800)	11 300 (9100 to 13 500)	11 100 (8980 to 13 400)	10 900 (8600 to 13 100)	10 500 (8200 to 12 800)	10 700 (8460 to 13 000)	10 300 (8010 to 12 700)
**Total outcomes**								
Incident tuberculosis	11 300 (9200 to 13 600)	11 300 (9200 to 13 600)	8860 (7060 to 11 000)	8210 (6530 to 10 200)	6390 (5150 to 7820)	5090 (4150 to 6200)	5680 (4600 to 7000)	4180 (3400 to 5090)
Incident rifampicin-susceptible tuberculosis	1980 (1400 to 2810)	1980 (1400 to 2810)	1440 (984 to 2160)	1440 (984 to 2160)	893 (624 to 1270)	893 (624 to 1270)	733 (514 to 1040)	733 (514 to 1040)
Incident multidrug-resistant or rifampicin-resistant tuberculosis	9310 (7400 to 11 500)	9310 (7400 to 11 500)	7410 (5730 to 9490)	6760 (5200 to 8780)	5500 (4260 to 6840)	4200 (3320 to 5260)	4940 (3820 to 6240)	3450 (2700 to 4290)
Incident tuberculosis deaths	2530 (2020 to 3120)	2530 (2020 to 3120)	1660 (1310 to 2070)	1420 (1130 to 1760)	1370 (1070 to 1720)	1040 (834 to 1280)	1290 (1010 to 1630)	936 (747 to 1160)
Prevalent tuberculosis deaths	3580 (3040 to 4130)	1230 (1020 to 1470)	1230 (1020 to 1470)	1230 (1020 to 1470)	1230 (1020 to 1470)	1230 (1020 to 1470)	1230 (1020 to 1470)	1230 (1020 to 1470)
**Incremental outcomes**								
Incident tuberculosis	Reference	0	−2440 (−3060 to −1900)	−3090 (−3880 to −2440)	−4900 (−6000 to −3950)	−6210 (−7500 to −5070)	−5620 (−6890 to −4540)	−7120 (−8610 to −5800)
Incident rifampicin-susceptible tuberculosis	Reference	0	−539 (−824 to −375)	−539 (−824 to −375)	−1090 (−1550 to −775)	−1090 (−1550 to −775)	−1250 (−1780 to −893)	−1250 (−1780 to −893)
Incident multidrug-resistant or rifampicin-resistant tuberculosis	Reference	0	−1900 (−2510 to −1410)	−2550 (−3290 to −1940)	−3810 (−4830 to −2960)	−5120 (−6360 to −4060)	−4370 (−5530 to −3370)	−5870 (−7280 to −4620)
Incident tuberculosis deaths	Reference	0	−871 (−1130 to −652)	−1110 (−1420 to −852)	−1160 (−1450 to −907)	−1490 (−1870 to −1180)	−1240 (−1540 to −970)	−1590 (−1980 to −1270)
Prevalent tuberculosis deaths	Reference	−2350 (−2790 to −1940)	−2350 (−2790 to −1940)	−2350 (−2790 to −1940)	−2350 (−2790 to −1940)	−2350 (−2790 to −1940)	−2350 (−2790 to −1940)	−2350 (−2790 to −1940)

Data are n (95% uncertainty interval). Reference indicates that other cells in the row are calculated as changes relative to this value. TPT=tuberculosis preventive therapy. HCM=household contact management.

* Fluoroquinolone refers to either levofloxacin or moxifloxacin.

**Figure 2:**
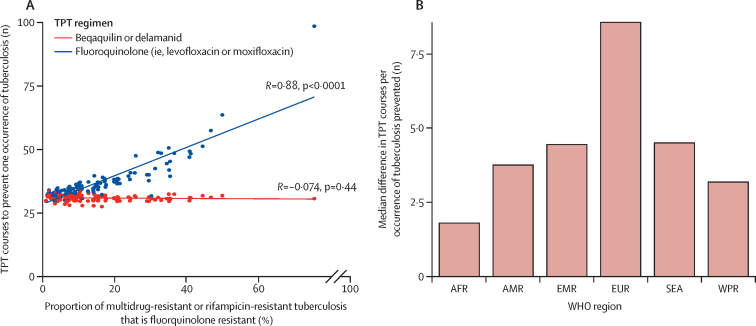
TPT courses required to prevent a tuberculosis episode Number of courses required to prevent a tuberculosis episode (A) as prevalence of fluoroquinolone resistance varies, comparing a bedaquiline or delamanid regimen with a levofloxacin or moxifloxacin regimen, where each circle represents the number of courses required to prevent a tuberculosis episode in a specific country, and (B) the median differences in the number of courses required in different WHO regions. High-income countries are excluded. TPT=tuberculosis preventive therapy. AFR=African region. AMR=region of the Americas. EMR=Eastern Mediterranean region. EUR=European region. SEA=South-East Asia region. WPR=Western Pacific region.

Without HCM, 11 300 contacts (95% UI 9200–13 600) younger than 15 years would have developed incident tuberculosis and 6110 contacts (5230–7100) younger than 15 years would have died (Table 1, Table 2). HCM without the provision of tuberculosis preventive therapy would not have reduced the number of incident cases but would have identified co-prevalent tuberculosis disease, resulting in 2350 fewer deaths (95% UI 1940–2790). Provision of tuberculosis preventive therapy would have reduced incident tuberculosis by up to 7120 cases (95% UI 5800–8610), dependent on the tuberculosis preventive therapy recipients and regimen, and would have averted up to a further 1590 deaths (1270–1980). Up to 5870 tuberculosis episodes (95% UI 4620–7280) averted would have been multidrug-resistant or rifampicin-resistant tuberculosis. As a result, between 24 tuberculosis preventive therapy courses (ie, delamanid or bedaquiline to children younger than 5 years or younger than 15 years with HIV) and 38 courses (ie, fluoroquinolone to all children) would have been needed to prevent a tuberculosis episode, and between 60 contacts (ie, delamanid or bedaquiline to all children) and 71 contacts (ie, fluoroquinolone to children younger than 5 years or younger than 15 years with HIV) would be screened per death averted (appendix p 23). Correspondingly, assuming no drug resistance, screening between 84 and 91 contacts was needed to avert one death (appendix pp 24–25). The number of fluoroquinolone-based tuberculosis preventive therapy courses that were needed to prevent tuberculosis increased with the prevalence of fluoroquinolone resistance in a given setting (figure 2).

**Table 2:** Cost-effectiveness for global household contact management interventions for multidrug-resistant and rifampicin-resistant tuberculosis in children younger than 15 years for 2019

	**No intervention**	**HCM only**	**HCM and TPT for all children aged <5 years or <15 years living with HIV**				**HCM and TPT for all children aged <5 years or <15 years living with HIV or with a positive tuberculin skin test**				**HCM and TPT for all children aged <15 years**			
			Levofloxacin	Moxifloxacin	Delamanid	Bedaquiline	Levofloxacin	Moxifloxacin	Delamanid	Bedaquiline	Levofloxacin	Moxifloxacin	Delamanid	Bedaquiline
Cost, US$ million	51 (31 to 80)	114 (86 to 151)	117 (90 to 153)	119 (91 to 154)	128 (100 to 164)	119 (92 to 154)	127 (100 to 162)	130 (103 to 166)	157 (128 to 194)	130 (103 to 164)	135 (108 to 171)	140 (113 to 177)	184 (152 to 223)	141 (112 to 174)
Deaths	6110 (5230 to 7100)	3760 (3130 to 4440)	2890 (2420 to 3410)	2890 (2420 to 3410)	2650 (2230 to 3100)	2650 (2230 to 3100)	2600 (2180 to 3080)	2600 (2180 to 3080)	2280 (1920 to 2660)	2280 (1920 to 2660)	2520 (2110 to 2980)	2520 (2110 to 2980)	2170 (1830–2530)	2170 (1830 to 2530)
Life-years lost, 3% discounted	171 000 (145 000 to 199 000)	105 000 (86 900 to 124 000)	80 600 (67 300 to 95 200)	80 600 (67 300 to 95 200)	73 800 (61 700 to 86 400)	73 800 (61 700 to 86 400)	72 600 (60 700 to 86 000)	72 600 (60 700 to 86 000)	63 500 (53 500 to 74 300)	63 500 (53 500 to 74 300)	70 300 (58 800 to 83 400)	70 300 (58 800 to 83 400)	60 400 (50 900–70 700)	60 400 (50 900 to 70 700)
Incremental cost, US$ million	Reference	63 (40 to 95)	66 (43 to 97)	68 (44 to 99)	77 (53 to 108)	68 (45 to 99)	76 (52 to 108)	79 (55 to 111)	106 (79 to 141)	79 (54 to 110)	84 (59 to 116)	89 (64 to 122)	133 (102 to 171)	90 (63 to 122)
Incremental deaths	Reference	−2350 (−2790 to −1940)	−3220 (−3840 to −2690)	−3220 (−3840 to −2690)	−3470 (−4150 to −2880)	−3470 (−4150 to −2880)	−3510 (−4170 to −2930)	−3510 (−4170 to −2930)	−3840 (−4550 to −3220)	−3840 (−4550 to −3220)	−3590 (−4250 to −3010)	−3590 (−4250 to −3010)	−3950 (−4660 to −3330)	−3950 (−4660 to −3330)
Incremental life-years saved, 3% discounted	Reference	65 700 (54 100 to 78 100)	90 100 (74 600 to 108 000)	90 100 (74 600 to 108 000)	96 900 (80 300 to 116 000)	96 900 (80 300 to 116 000)	98 000 (81 700 to 117 000)	98 000 (81 700 to 117 000)	107 000 (89 600–128 000)	107 000 (89 600 to 128 000)	100 000 (83 800 to 119 000)	100 000 (83 800 to 119 000)	110 000 (92 600 to 131 000)	110 000 (92 600 to 131 000)
ICER, US$ per DALY	..	960	738	754	799	703	773	807	992	737	838	890	1208	814

**Figure 3:**
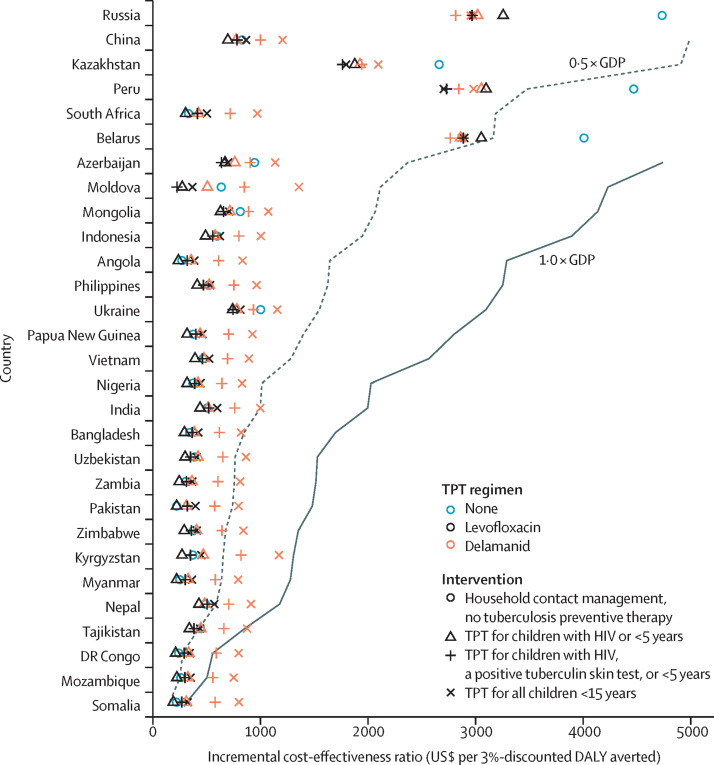
Incremental cost-effectiveness ratios for household contact management Ratios for settings with different GDP per capita for the 30 countries with a high burden of multidrug-resistant or rifampicin-resistant tuberculosis in 2019, as classified by WHO. Lower incremental cost-effectiveness ratios are more cost-effective. US$ are 2020 US$. TPT=tuberculosis preventive therapy. DALY=disability-adjusted life-year. GDP=gross domestic product per year.

Data are n (95% uncertainty interval) unless otherwise stated. Reference indicates that other cells in the row are calculated as changes relative to this value. US$ are 2020 US$. Global ICERs are for summary comparisons between interventions (figure 3; appendix pp 26–27). DALY=disability-adjusted life-year. ICER=incremental cost-effectiveness ratio. TPT=tuberculosis preventive therapy. HCM=household contact management.

Globally, the cost of tuberculosis treatment among child contacts in the absence of HCM and tuberculosis preventive therapy was estimated to be US$51 million (95% UI $31–80 million), whereas the cost under universal HCM without tuberculosis preventive therapy would have been $114 million ($86–151 million). The cost under HCM with provision of tuberculosis preventive therapy to children younger than 5 years and those younger than 15 years living with HIV was up to $128 million (95% UI $100–164 million), depending on the regimen. The cost for additional provision of tuberculosis preventive therapy to children younger than 15 years with a positive TST was up to $157 million (95% UI $128–194 million), and the cost for provision to all contacts younger than 15 years was up to $184 million ($152–223 million; table 2). Globally, ICERs for HCM strategies varied between $703 and $1208 per DALY averted (table 2) and summarise interventions' relative cost-effectiveness in most settings. In the 30 countries with a high burden of multidrug-resistant or rifampicin-resistant tuberculosis, as classified by WHO, most strategies would be considered cost-effective judged against a threshold of 0·5 × gross domestic product per capita (levofloxacin and delamanid, the regimens currently under trial, are shown in figure 3), with fluoroquinolone-based tuberculosis preventive therapy strategies typically having similar ICERs to screening only, and delamanid-containing strategies being less cost-effective as the number of children treated within a strategy increases. For interventions with tuberculosis preventive therapy, ICERs usually decreased with decreasing size of the tuberculosis preventive therapy recipient group. For some countries, HCM with tuberculosis preventive therapy was markedly more cost-effective than HCM alone, and delamanid had similar ICERs to levofloxacin. Other countries are shown in the appendix (p 26). Assuming that not all index patients had pulmonary disease made little difference (appendix p 28). Discount rates of 5% per year increased ICERs by a mean factor of 1·52, whereas 1% per year discounting reduced ICERs by a mean factor of 0·57 compared with 3% per year discounting (appendix pp 32–34).

## Discussion

This study estimates the effect and cost-effectiveness of HCM for children exposed to multidrug-resistant or rifampicin-resistant tuberculosis. Most deaths were prevented by the identification and treatment of co-prevalent tuberculosis, but tuberculosis preventive therapy was effective in preventing incident tuberculosis and associated deaths, and most strategies in most countries were cost-effective. Although the greatest number of cases and deaths were prevented when giving tuberculosis preventive therapy to all children younger than 15 years, more targeted strategies were more cost-effective, particularly when using delamanid-based or bedaquiline-based regimens. Levofloxacin was more cost-effective than was delamanid in almost all countries, but delamanid and bedaquiline can have an important role in tuberculosis preventive therapy where fluoroquinolone resistance is common, especially if prices decline. Compared with HCM for patients with rifampicin-susceptible tuberculosis, HCM for patients with multidrug-resistant or rifampicin-resistant tuberculosis required substantially less effort to prevent each death, suggesting that it should be seen as a particularly high priority.

Evidence relevant specifically to the effect and cost-effectiveness of HCM for patients with multidrug-resistant or rifampicin-resistant tuberculosis is scarce. HCM for index patients with drug-susceptible tuberculosis was evaluated with a large community trial in Vietnam, which found that active screening of household contacts at baseline and follow-up led to significantly more people diagnosed with tuberculosis than under the standard of care.^20^ Although Shah and colleagues did a systematic review of the yield of contact investigations in households of patients with drug-resistant tuberculosis,^10^ no studies have measured the effect of HCM for patients with drug-resistant tuberculosis. Marks and colleagues did a systematic review of the efficacy of multidrug-resistant tuberculosis preventive therapy and identified six studies that pointed to a good efficacy and cost-effectiveness among household contacts of patients with multidrug-resistant tuberculosis.^21^ Fox and colleagues also undertook a cost-effectiveness analysis of fluoroquinolone tuberculosis preventive therapy for contacts of patients with multidrug-resistant tuberculosis in the USA and found it to be highly cost-effective, reducing costs, incident multidrug-resistant tuberculosis, and death.^22^

In addition to showing the effect of tuberculosis preventive therapy delivery, our findings emphasise the importance of the case-finding component of HCM in averting paediatric tuberculosis deaths. In the absence of tuberculosis preventive therapy, case-finding averted 2350 tuberculosis deaths (95% UI 1940–2790), and in the most comprehensive tuberculosis preventive therapy scenario (ie, bedaquiline or delamanid provided to all children <15 years) an additional 1590 tuberculosis deaths (95% UI 1270–1980) were averted. Furthermore, we did not consider the benefits from screening adults for tuberculosis. The estimated effect of HCM without preventive therapy shows that, even where national tuberculosis programmes are unable to provide tuberculosis preventive therapy, screening of household contacts alone is a crucial intervention that has a strong evidence base, is included in almost all guidance, and could be rapidly scaled up.

The effect of tuberculosis preventive therapy on incident tuberculosis and deaths averted increased as the population of recipients expanded; providing tuberculosis preventive therapy to all children younger than 15 years had the greatest effect. However, the incremental benefit of providing additional tuberculosis preventive therapy decreased as the number of recipients increased. More tuberculosis preventive therapy courses were required per incident tuberculosis episode and tuberculosis death averted, although fewer household contacts were required to be screened because the number of contacts screened did not change, so the benefit of screening each contact was higher. As a result, the cost-effectiveness decreased as the recipients increased. The pattern of increased cost-effectiveness when concentrating preventive therapy on people with highest risk of progression to disease provides additional motivation for the availability of cheap, easy-to-use tests of infection at point of care and tests to predict future disease progression.^23^

The effect of tuberculosis preventive therapy with delamanid or bedaquiline (assumed here to have identical efficacy) in all contacts younger than 15 years was higher than the effect of tuberculosis preventive therapy with a fluoroquinolone, reducing tuberculosis incidence by 7120 cases (95% UI 5800–8610) compared with by 5620 cases (4540–6890) and deaths due to incident tuberculosis by 1590 deaths (1270–1980) compared with by 1240 deaths (970–1540). As a result, the number of tuberculosis preventive therapy courses required and household contacts screened per death averted were lower. However, the higher drug costs meant that delamanid was less cost-effective than levofloxacin (ie, $1208 *vs* $838 per DALY averted if tuberculosis preventive therapy was provided to all children younger than 15 years). The range in ICERs across different tuberculosis preventive therapy recipient groups was larger for delamanid and bedaquiline than for the fluoroquinolones, as the drug costs formed a larger proportion of the overall cost, which provides motivation for lowering prices of novel drugs.

For most interventions in most settings, HCM for patients with multidrug-resistant or rifampicin-resistant tuberculosis would be judged to be cost-effective against a threshold of 0·5 × gross domestic product. It is not the role of analysts to specify cost-effectiveness thresholds; 0·5 × gross domestic product is reported as a crude summary of econometric work to estimate typical marginal ICERs in health systems. Cost-effectiveness acceptability curves (appendix p 27) provide decision makers with probabilities of interventions being cost-effective at their preferred threshold. The benefit of delamanid or bedaquiline over a fluoroquinolone increased considerably in settings with high prevalence of fluoroquinolone resistance, such as many countries in the WHO European Region, with potentially four times as many fluoroquinolone-based tuberculosis preventive therapy courses required per tuberculosis episode averted. Russia, Belarus, Kazakhstan, and Peru had unusual ICER rankings, driven by exceptionally high treatment costs for patients with multidrug-resistant or rifampicin-resistant tuberculosis.

Our absolute results could be considered an upper bound on the benefits of HCM for patients with multidrug-resistant or rifampicin-resistant tuberculosis, since we assume a maximal intervention coverage, which might not be achievable in practice, and a comparator with no HCM. However, we do not evaluate transmission reductions, which would increase intervention benefits and cost-effectiveness. Similarly, we do not include benefits from screening adult household contacts. Our assumption of maximal treatment coverage will also affect cost-effectiveness estimates less than estimates of total treatment benefits. We chose 2019 as the most recent year before the COVID-19 pandemic; however, a probable increase in household transmission of *M tuberculosis* during the pandemic could enhance the potential intervention effect.^24^ We assumed that tuberculosis preventive therapy had no efficacy against resistant organisms. In reality, resistance is not binary, and first-line drugs will probably have some efficacy against multidrug-resistant tuberculosis strains, as has been reported in a study in Peru,^25^ and as will fluoroquinolones against fluoroquinolone-resistant organisms. Many of the parameters we used in the model were derived from the paediatric drug-susceptible tuberculosis literature, and an important consideration when modelling HCM strategies for multidrug-resistant or rifampicin-resistant tuberculosis is the fitness of drug-resistant strains. Kodama and colleagues^26^ reviewed the relative infectiousness and virulence of drug-resistant compared with drug-susceptible *M tuberculosis* strains, noting that contacts of patients with drug-resistant tuberculosis were more likely to be infected, but no more likely to have tuberculosis disease. Finally, our study was limited by missing data in some countries, including for fluoroquinolone resistance and unit costs, necessitating statistical modelling and prediction entailing substantial uncertainty (appendix pp 5, 21).

The upcoming trials for multidrug-resistant or rifampicin-resistant tuberculosis tuberculosis preventive therapy will provide valuable information on not only the efficacy of tuberculosis preventive therapy regimens and their acceptability but also their associated costs, toxicity, and other unintended consequences. This evidence should be included in updated analyses. It will be important to assess the potential for different regimens to drive resistance that might compromise multidrug-resistant or rifampicin-resistant tuberculosis treatment options. Although there is no evidence that tuberculosis preventive therapy for patients with rifampicin-susceptible tuberculosis drives resistance,^27^ widely used tuberculosis preventive therapy without robust exclusion of disease could promote acquired resistance. Moreover, widespread use of fluoroquinolones could generate fluoroquinolone resistance in bacteria other than *M tuberculosis*, potentially compromising the treatment of life-threatening infections. The effect of prolonged use of broad-spectrum antibiotics on the microbiome also needs careful evaluation and consideration.^28^ The potential for promoting resistance emphasises the importance of further analyses focused on antimicrobial resistance to appropriately evaluate the use of tuberculosis preventive therapy,^29^ both in terms of averting future transmission and the effects of the treatment on other bacteria, and in terms of further resistance acquisition and the associated costs, such as the need to develop new antibiotics. Further analyses focused on antimicrobial resistance are particularly relevant given current debates around stewardship of new tuberculosis drugs, and the emergence of resistance to these, which could increase the comparative cost of their use for tuberculosis preventive therapy. The decision to implement HCM for patients with multidrug-resistant or rifampicin-resistant tuberculosis in each setting will depend on the cost of contact screening; the efficacy, adverse events, acceptability, and costs associated with the regimens in use; the population demographic and resistance profiles; and the resources available. Our model provides a framework for that decision making, showing that HCM for patients with multidrug-resistant or rifampicin-resistant tuberculosis is cost-effective in most settings. Widespread use of HCM for patients with multidrug-resistant or rifampicin-resistant tuberculosis could avert up to 6000 multidrug-resistant or rifampicin-resistant tuberculosis episodes in children. In the context of an estimated annual 30 000 episodes of multidrug-resistant tuberculosis in children, HCM could avert a substantial proportion of multidrug-resistant or rifampicin-resistant tuberculosis cases and deaths in children globally.

## Data sharing

All data and code are publicly available at https://github.com/petedodd/DRHHCM.

## Declaration of interests

We declare no competing interests.
