# Supplementary material for: The global impact of household contact management for children on multidrug-resistant and rifampicin-resistant tuberculosis cases, deaths, and health-system costs in 2019: a modelling study
Source: Lancet Glob Health. 2022 May 18;10(7):e1034–44. doi: 10.1016/S2214-109X(22)00113-9 (PMC9197775; doi:10.1016/S2214-109X(22)00113-9)
Supplement: Supplementary appendix [file mmc1.pdf]

THE LANCET
Global Health

Supplementary appendix

This appendix formed part of the original submission and has been peer reviewed. We post it as supplied by the authors.

Supplement to: Dodd PJ, Mafirakureva N, Seddon JA, McQuaid CF. The global impact of household contact management for children on multidrug-resistant and rifampicin-resistant tuberculosis cases, deaths, and health-system costs in 2019: a modelling study. *Lancet Glob Health* 2022; published online May 18. https://doi.org/10.1016/S2214-109X(22)00113-9.

The impact and cost-effectiveness of multidrug- and rifampicin-resistant tuberculosis household contact management in children: a modelling study (Appendix)

Peter J Dodd, Nyashadzaishe Mafirakureva, James A Seddon, and Christopher F McQuaid

Supplementary methods: epidemiological & outcomes model **2**
Index case drug-resistance type & number of household contacts 2
Number of contacts 2
Rifampicin resistance and HIV status 2
Prevalence of symptoms among contacts 2
Prevalence of fluoroquinolone resistance 3
Risks of infection, co-prevalent tuberculosis & incident tuberculosis 5
Latent tuberculosis infection prevalence 5
Co-prevalence of tuberculosis disease 6
Incident tuberculosis risk & concordance of drug-resistance type 6
Efficacy of preventive therapy & adverse events 6
Case detection for tuberculosis 7
Detection of all tuberculosis 7
Detection of MDR/RR tuberculosis 8
Treatment outcomes 8
Parameter distributions used 8
Computational approach 12
Modelling approach & uncertainty quantification 12
Metrics calculated 13
Reproducibility 13

Supplementary methods: costing **13**
Resource use 13
Unit costs 15
Sources and assumptions 15
Estimation of tuberculosis disease treatment costs 19
Estimates of implied cost-effectiveness thresholds and GDP per capita 21

Supplementary results **23**
Resources per case or death averted 23
Comparison to previous work 24
Additional health economic outputs 25
Sensitivity analysis around the fraction of MDR/RR-TB that is pulmonary 28
Sensitivity analysis around discount rate 32
Adverse event estimates 34
List of countries included 35

References **36**

Supplementary methods: epidemiological & outcomes model

Index case drug-resistance type & number of household contacts

Number of contacts

Our model for the number of household contact of tuberculosis cases uses a regression model developed in Dodd and colleagues,[1] that uses Demographic and Health Systems (DHS) surveys to predict the number of household members aged 0-4 years and 5-14 years for an adult of given sex and age category in each country. In that work we also investigated whether household structure was substantially different for tuberculosis patients compared with the general population, but failed to identify systematic differences. Here, we also assume that the number of household contacts of tuberculosis patients is not different depending on drug-resistance type.

World Health Organization (WHO) notification data were used with age- and sex-patterns for patients >=15 years based on each country's most recent data. Where countries did not have age- and sex-stratified notification data, regional average patterns of the proportions were applied to total country notifications. In our main analysis, we assumed that all diagnosed MDR/RR-TB in adults was pulmonary, since diagnosis of drug resistance commonly relies on bacteriological specimens, most often samples of pulmonary secretions. However, we ran a sensitivity analysis (see below) that considered restricting the number of index cases in the same way as the all-TB analysis in Dodd and colleagues.[1]

Rifampicin resistance and HIV status

The number of contacts per tuberculosis patient from the above procedure is applied to the number of notified multidrug-resistant or rifampicin-resistant tuberculosis (MDR/RR-TB) cases reported to WHO for each country. The HIV prevalence among tuberculosis patients in each country was taken as the WHO estimate of this quantity. The antiretroviral therapy (ART) coverage in this group was taken as the fraction of notified tuberculosis patients with HIV in the WHO data who were on ART. The HIV prevalence among 0-4 year old and 5-14 year old contacts of tuberculosis/HIV index patients was based on a Ugandan cohort described in Martinez and colleagues[2] using beta distributions. The same proportion of child contacts living with HIV were assumed to be on ART as among adults living with HIV notified with tuberculosis.

Prevalence of symptoms among contacts

In costing the interventions, it is necessary to know what proportion of child household contacts would be found positive on a symptom-screen and therefore be considered to have presumptive tuberculosis, as this population would then require further evaluation for tuberculosis. Martinez and colleagues in a Ugandan cohort of household contacts <16 years found 21% of 1,718 were

symptomatic with WHO definitions. Sayedi and colleagues[3] in Afghanistan reported 20.1% of 117,643 household contacts under 5 were classed as presumptive tuberculosis.
Schwoebel and colleagues found that of 1,965 household contacts under 5 in various African cities, 43% reported cough and 30% with fever within 4 weeks. These data are difficult to synthesise, especially given differing symptom definitions. Given the similar point estimates from Sayedi and colleagues[3] and Martinez and colleagues[2] however, we adopt a value of 20% (+/- 10% relative uncertainty), modelled with a beta distribution.

Prevalence of fluoroquinolone resistance

Estimates of the proportion of rifampicin-resistant tuberculosis that is fluoroquinolone-resistant in each country are based on a modified method from a previous analysis of WHO-collated data among MDR-TB patients (Dodd and colleagues[4]). We used WHO-collated data on the number of patients with fluoroquinolone resistance (aggregate count *k*) from among those with rifampicin (aggregate count *N*) resistant tuberculosis in each country, and used the following 3 step procedure:

1. If data were available for a country, we drew 1,000 samples from a beta distribution *B(1+k,1+N-k)*, corresponding to a Bayesian posterior for a binomial model with uninformative (conjugate) *U(0,1)* prior.
2. If data were not available for a country but >=2 of the country's "neighbours" (see below) had data, we drew 1,000 samples by randomly sampling a neighbour with data and drawing from the neighbour's posterior defined by step 1 above.
3. If data were not available for a country or >=2 of the country's neighbours, we drew 1,000 samples by randomly sampling a country with data from the same WHO region and drawing from its posterior defined by step 1 above.

The notion of "neighbour" used in this approach was defined by constructing a modified nearest-5-neighbours adjacency structure. The initially-generated nearest-5-neighbours adjacency structure had some features that are undesirable. In particular, the network is not connected (i.e. splits into multiple parts that can't be reached from each other by following links), and Russia is not connected to all former Soviet republics (whereas shared political history and borders mean similarities are likely). To address these issues, we introduced transatlantic links based on flight paths carrying more than 900K passengers per year. We also linked the South Pacific islands WLF and TON to FJI. Finally, we linked RUS to EST, LVA, UKR, GEO and AZE, motivated by geographical proximity and historical integration. The adjacency structure is shown in Figure 1.

The results of the sampling procedure are shown in Figure 2, with panels for each WHO region (AFR=African Region; AMR=American Region; EUR=European Region; EMR=Eastern Mediterranean Region; SEA=South-East Asia Region; WPR=Western Pacific Region), and error bars showing the median and interquartile range for each country's sample. We generated estimates for 215 counties. Mean regional prevalence of fluoroquinolone resistance in MDR/RR-TB ranged from 11% in AFR to 29% in EUR.

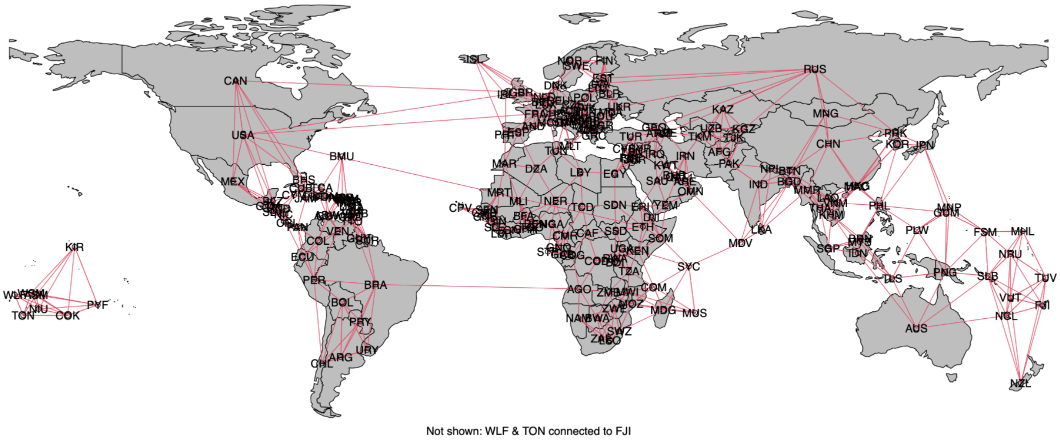

Figure 1 Neighbour structure used in the sampling procedure (not all links shown)

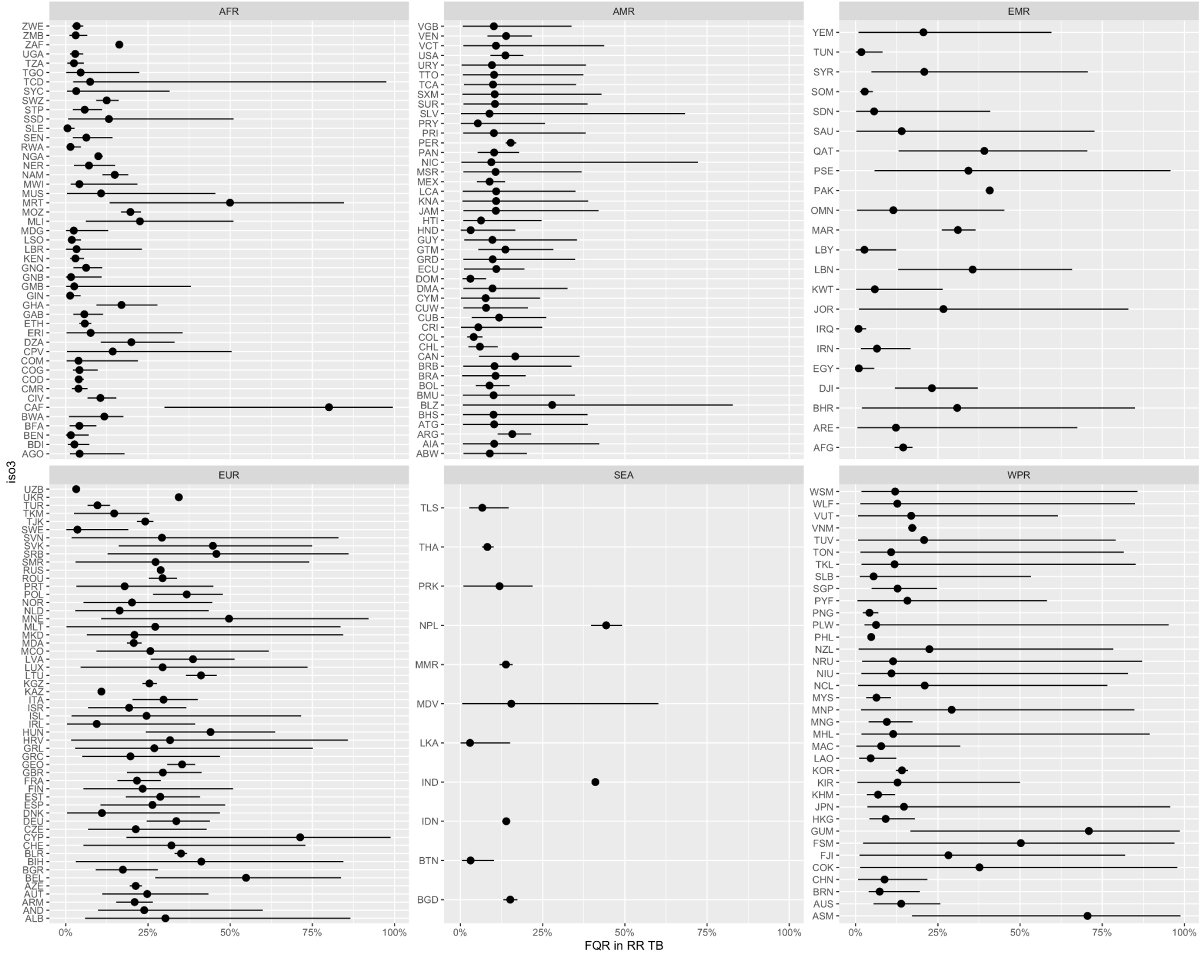

Figure 2 Fluoroquinolone resistance in MDR/RR-TB, grouped by WHO region

Risks of infection, co-prevalent tuberculosis & incident tuberculosis

Latent tuberculosis infection prevalence

In their systematic review and meta-analysis of household contact investigations for drug-resistant index cases, Shah and colleagues found 47.2% (0.0% - 61.4%) prevalence of latent tuberculosis infection (LTBI), but were not able to present data by age. Most people included in this data were living in low- and middle-income countries (LMICs). In the systematic review and meta-analysis of Fox and colleagues[5] for all tuberculosis (drug-resistant and drug-susceptible), the prevalence of LTBI among contacts of all ages in LMICs was estimated as 51% (47.1% - 55.8%). Since this is comparable with the estimate of Shah and colleagues, we continue to use the age-stratified LTBI prevalence estimates from Fox and colleagues: 35.5% (30.3% - 42.1%) for 0-4 year olds and 53.1% (42.0% - 63.9%) for 5-14 year olds in LMICs; 16.3% (9.2% - 27.0%) for 0-4 year olds and 18.4% (11.8% - 27.5%) for 5-14 year olds in high-income countries.

Co-prevalence of tuberculosis disease

Fox and colleagues found a summary co-prevalence of tuberculosis disease across all ages of 3.1% (2.2% – 4.4%) for LMICs; lower than the summary estimate of Shah and colleagues[6] for drug-resistant tuberculosis, i.e. 7.8% (5.6% - 10.0%). It may be the case that longer delays to care-seeking or other factors mean that co-prevalence rates are higher among contacts of drug-resistant than drug-susceptible tuberculosis. However, Fox and colleagues also reported LMIC summary estimates for co-prevalent tuberculosis of 10.0% (5.0% – 18.9%) in children under 5 years, and 8.4% (2.8% – 22.6%) for children aged 5-14 years. Rather than try to adjust the age-specific co-prevalence estimates from Fox and colleagues we used in our previous work,[1] we here use the single co-prevalence reported in Shah and colleagues for both age groups.

Incident tuberculosis risk & concordance of drug-resistance type

Since our previous study,[1] a large individual-patient systematic review and meta-analysis of progression risks among children after close exposure to tuberculosis has been published by Martinez and colleagues.[7] We use the 2 year progression risks from Martinez and colleagues for children with LTBI, for age groups 0-4 years and 5-14 years as risk of progression within 1 year (which most progression in relative age groups is). We assume no difference in progression risks by drug-resistance type. Progression risks were applied to the population of household contacts with LTBI less those with co-prevalent active disease. Progression to incident tuberculosis was assumed more likely in children living with HIV (depending on ART status), based on rate ratios from a systematic review and meta-analysis.[8]

Not all co-prevalent or incident tuberculosis will have drug-resistance types matching that of the index case. To account for imperfect concordance between household index cases and other household disease, we make use of the systematic review and meta-analysis of Chiang and colleagues[9] which found a pooled concordance of 82.6% (72.3% - 90.9%) isoniazid/rifampicin concordance comparing index cases with cases among household contacts. We assume that this probability, p, of being concordant with the index case means that a fraction $(1 - p)$ household contacts have rifampicin-susceptible tuberculosis. Of contacts who are concordant for rifampicin resistance, we assume that a fraction of them are fluoroquinolone resistant based on the method from Dodd and colleagues,[4] described above. Note that the fluoroquinolone resistance status of the index case is not modelled, nor is fluoroquinolone resistance among rifampicin-susceptible tuberculosis considered. While it is not possible to test the drug-resistance type of LTBI, we assume that this parameter governs LTBI in the same way, so that any incident tuberculosis has the same patterns of concordance with the index case.

Efficacy of preventive therapy & adverse events

While the efficacy of preventive therapy regimens for MDR/RR-TB are currently under evaluation, we assume that all preventive therapy regimens that are appropriate to the drug-resistance type of the LTBI have the same efficacy in reducing tuberculosis incidence as isoniazid preventive therapy has

for drug-susceptible LTBI. Where the preventive therapy regimen was not appropriate to the drug-resistant type of the LTBI we assumed an efficacy of zero.

Since our previous analysis,[1] which used the systematic review and meta-analysis for children Ayieko and colleagues,[10] Martinez and colleagues[7] have published meta-analytic estimates of preventive therapy efficacy in children that are based on a much larger number of children, and which accord with the older Cochrane review estimates of isoniazid preventive therapy efficacy in adults of Smieja and colleagues.[11] We therefore use the estimates in Martinez and colleagues[7] (stratified by tuberculin skin test [TST] status for use in interventions that employ TST criteria). For children living with HIV, we used the efficacy reported for isoniazid preventive therapy in children living with HIV by Zunza and colleagues[12] in ART-naive children.

Very little data has been reported on adverse events (AEs) in children under MDR/RR-TB preventive therapy. Seddon and colleagues[13] report 6 grade 3 or 4 AEs and 20 grade 2 AEs from 186 children aged 14-47 months receiving ofloxacin, ethambutol, and high-dose isoniazid for 6 months. Malik and colleagues[14] reported 11 grade 2 AEs and 0 grade 3 or 4 AEs from 172 contacts with a median age 7 years receiving a fluoroquinolone-containing MDR/RR-TB preventive therapy regimen. Grade 2 AEs are not stratified by age, but patients receiving preventive therapy were aged 15 or younger. We group grade 3 and 4 AEs as serious, requiring hospitalization; we consider grade 2 AEs as requiring an outpatient treatment; we ignore grade 1 AEs. We parameterized the proportion of children experiencing AEs or severe AEs based on the pooled counts across Seddon and colleagues and Malik and colleagues.

Case detection for tuberculosis

Detection of all tuberculosis

While all index patients in our model have MDR/RR-TB, imperfect concordance of drug resistance type means the overall tuberculosis detection is still a necessary input. Detection of MDR/RR-TB (see below) is used together with the overall case detection ratio (CDR) to compute the conditional probability that given tuberculosis in a child is detected, it will be correctly identified as MDR/RR-TB. The population CDR for all tuberculosis in those 0-4 years or 5-14 years was based on the ratio of age-stratified notification and WHO incidence estimates, modelled with beta distributions matched to the mean and variance. Following previous work,[1] we hypothesized that the CDR (for either co-prevalent or incident tuberculosis) among children cohabiting with patients with diagnosed tuberculosis would be higher than average, and therefore allowed this to range (with uniform distribution) between the population CDR as a lower bound and 1.5 x this (or 1 if smaller) as an upper bound. The motivation for the factor of 1.5 is described in Dodd and colleagues,[1] and is based on assuming that all notifications among children are among those who cohabit with a diagnosed tuberculosis patient. Introduction of this factor leads to conservative estimates of intervention impact by strengthening the counterfactual detection under standard of care. Under household contact management interventions, we assume that the CDR is 1 (all children with tuberculosis detected), which corresponds to the use of operational data for co-prevalence estimates, which factor in imperfections in screening and diagnostic sensitivity under realistic deployment. Detection (modelled via CDR) was identified with receiving treatment for tuberculosis.

Detection of MDR/RR tuberculosis

We calculated case detection ratios specific for MDR/RR-TB by age 0-4 years and 5-14 years based on WHO-reported number of new or relapse notified cases by age, weighted by the proportion of MDR/RR-TB patients aged 0-14 years who were started on treatment compared to the estimated MDR/RR-TB incidence in 0-4 and 5-14 year olds. The incident estimates were calculated using the estimated incidence of all tuberculosis in 0-4 and 5-14 year olds weighted by the estimated percentage of MDR/RR-TB occurring in new cases. The number of adults treated was calculated using the number of confirmed and unconfirmed cases started on treatment for MDR/RR-TB, less the number of 0-14 year olds started on treatment. Where the number of 0-14 year olds started on treatment for MDR/RR-TB was not available, we assumed that all 0-14 year olds diagnosed with MDR/RR-TB were started on treatment. Where this was not available we assumed that no 0-14 year olds were treated for MDR/RR-TB. In the model, the MDR/RR-TB CDR was used together with the all-tuberculosis CDR in each country and age group to compute a probability of receiving correct treatment for MDR/RR-TB. Those diagnosed but not correctly diagnosed with MDR/RR-TB were assumed to receive ineffective first-line treatment for their tuberculosis. Under interventions, all children with MDR/RR-TB who are detected are assumed to receive appropriate treatment.

Treatment outcomes

We used case fatality ratios (CFRs) to model death or survival following a tuberculosis episode. CFRs for untreated tuberculosis were based on the systematic review and meta-analysis of Jenkins and colleagues;[15] as in previous work, we use their summary estimates of the pre-chemotherapy era outcomes as a proxy for untreated outcomes, and stratify these by age 0-4 years and 5-14 years. Jenkins and colleagues also provide estimates of CFRs on tuberculosis treatment, which we use for second-line treatment of rifampicin-susceptible tuberculosis. The influence of HIV/ART status is also captured following the approach described in Dodd and colleagues,[16] which used expert elicitation in the case of untreated tuberculosis. The systematic review and individual-patient meta-analysis of Harausz and colleagues[17] provide estimates of CFRs for second-line treatment of children with MDR-TB, reporting 92% (86%-96%) of bacteriologically confirmed patients had successful treatment outcomes; we apply these estimates to correctly treated MDR/RR-TB. For children with MDR/RR-TB who only receive first-line treatment, we assume the same CFRs as if they were untreated.

Parameter distributions used

The Table 1 shows all parameters used in the model together with the distribution used to model their uncertainty. Figure 3 shows these distributions graphically.

Table 1 Model parameters and their distributions

NAME	DISTRIBUTION	DESCRIPTION	SOURCE	MEAN (IQR)
ontxY	LN(-3.963316,0.6457913)	CFR children <5 on TB treatment	Jenkins et al 2017	0.019 (0.012 - 0.029)
ontxO	LN(-4.828314,0.4817445)	CFR children 5-14 on TB treatment	Jenkins et al 2017	0.008 (0.006 - 0.011)

hivartOR:mn	MVN: [2.6375681, -0.5683867]	ORs of death on TB treatment, (OR HIV+ vs -) x (ART -/+): mean	Jenkins et al 2017, Dodd et al 2017	
hivartOR:sg	MVN: [[0.2325509,-0.2325509],[-0.2325509,0.6367345]]	ORs of death on TB treatment, (OR HIV+ vs -) x (ART -/+): variance	Jenkins et al 2017, Dodd et al 2017	
notxY	LN(-0.830113,0.08035318)	CFR children <5 without TB treatment	Jenkins et al 2017	0.436 (0.413 - 0.460)
notxO	LN(-1.903809,0.1285165)	CFR children 5-14 without TB treatment	Jenkins et al 2017	0.149 (0.137 - 0.162)
notxHY	B(77.13050,11.10817)	CFR children <5 without TB treatment (HIV+/ART-)	Dodd et al 2017	0.877 (0.852 - 0.899)
notxHO	B(19.59083,6.89700)	CFR children 5-14 without TB treatment (HIV+/ART-)	Dodd et al 2017	0.746 (0.686 - 0.800)
notxHAY	B(15.18683,12.87500)	CFR children <5 without TB treatment (HIV+/ART+)	Dodd et al 2017	0.542 (0.478 - 0.605)
notxHAO	B(10.43383,11.08417)	CFR children 5-14 without TB treatment (HIV+/ART+)	Dodd et al 2017	0.484 (0.412 - 0.558)
hivpi	LN(2.066863,0.2800718)	IRR for TB incidence given HIV+/ART- (for individuals)	Dodd et al 2017	7.900 (6.540 - 9.543)
artp	LN(-1.203973,0.150482)	HR for TB incidence given HIV+/ART+ vs HIV+/ART-	Dodd et al 2016	0.300 (0.271 - 0.332)
HHhivprev04	B(55,526)	Prevalence of HIV in child HH contacts of HIV+ index case	Martinez et al 2017	0.094 (0.086 - 0.103)
HHhivprev514	B(54,854)	Prevalence of HIV in child HH contacts of HIV+ index case	Martinez et al 2017	0.059 (0.054 - 0.065)
LTBI04	B(106.7330582,193.9234438)	LTBI prevalence	Fox et al 2013	0.355 (0.336 - 0.373)
LTBI514	B(41.83776346,36.95275153)	LTBI prevalence	Fox et al 2013	0.531 (0.493 - 0.569)
LTBI04hi	B(10.62231,54.54526)	LTBI prevalence	Fox et al 2013	0.160 (0.131 - 0.192)
LTBI514hi	B(17.0386,75.56247)	LTBI prevalence	Fox et al 2013	0.182 (0.156 - 0.210)
iptRR	B(45.48834,77.45311)	RR for incident TB given IPT, age <15	Martinez et al 2018	0.369 (0.340 - 0.399)
iptRRtstpos	B(11.23657,113.6142)	RR for incident TB given IPT in TST+, age <15	Martinez et al 2018	0.088 (0.072 - 0.106)
iptRRhivpos	LN(-1.171183,0.5127492)	RR for incident TB given IPT in HIV+, age <15	Zunza et al 2017	0.310 (0.219 - 0.438)
CFRrtx.RR	B(11.09004,127.5355)	CFR RR-ATT when truly RR	Harausz et al 2018	0.078 (0.064 - 0.094)
coprevDRkids	LN(2.054124,0.1417502)	coprevalence in percent of HH contacts of DRTB	Shah et al 2017	7.800 (7.089 - 8.583)
concord	B(57.40241,12.09203)	concordance in DR type	Chiang et al 2020	0.829 (0.797 - 0.858)
prog04	B(5.152793,21.96717)	LTBI+ progression u5	Martinez et al 2018	0.182 (0.136 - 0.236)
prog514	B(4.151282,43.02238)	LTBI+ progression u5	Martinez et al 2018	0.082 (0.058 -

				0.112)
fracSymptomatic	B(2.936,11.744)	Fraction of contacts presumptive TB	Martinez 2017 & Sayedi 2020	0.186 (0.124 - 0.262)
fracAE	B(31,358)	Fraction of PT with G2 AEs	Malik 2020 & Seddon 2013	0.079 (0.070 - 0.089)
fracSAE	B(6,358)	Fraction of PT with G3/4 AEs	Malik 2020 & Seddon 2013	0.016 (0.012 - 0.020)

CFR: case fatality rate; TB: tuberculosis; OR: odds ratio; HR: hazard ratio; HIV: human immunodeficiency virus; HH: household; ART: antiretroviral therapy; RR: rifampicin-resistant; DR: drug-resistant(ce); ATT: antituberculosis treatment; AE: adverse event; Gn: grade n; IPT: isoniazid preventive therapy; LTBI: latent tuberculosis infection; B: beta distribution; LN: log-normal distribution; MVN: multivariate normal distribution

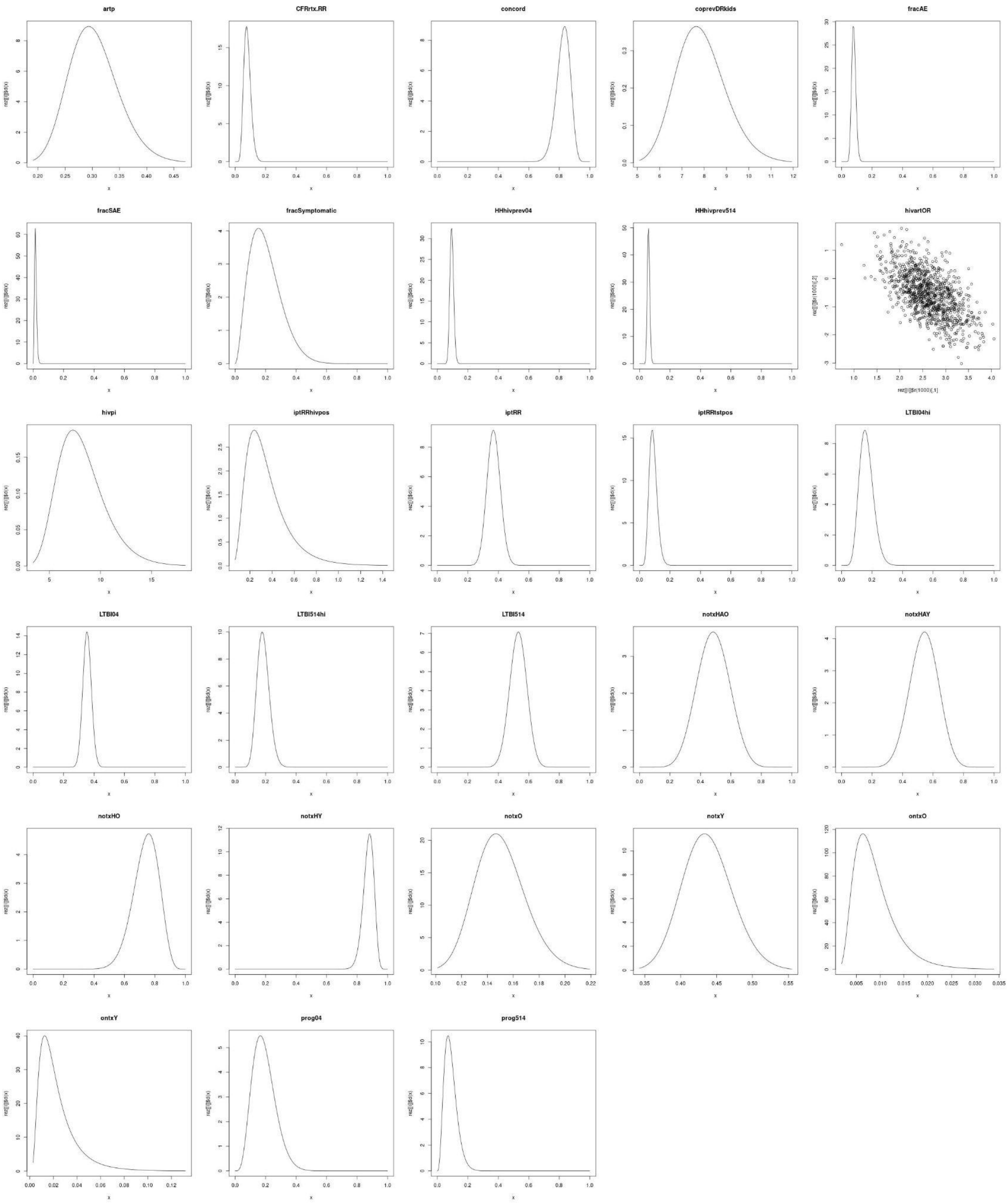

Figure 3 Probability density functions for model parameters. (“hivartOR” is a scatter plot because two parameters are modelled with a bivariate distribution. See Dodd et al for details.[16])

Computational approach

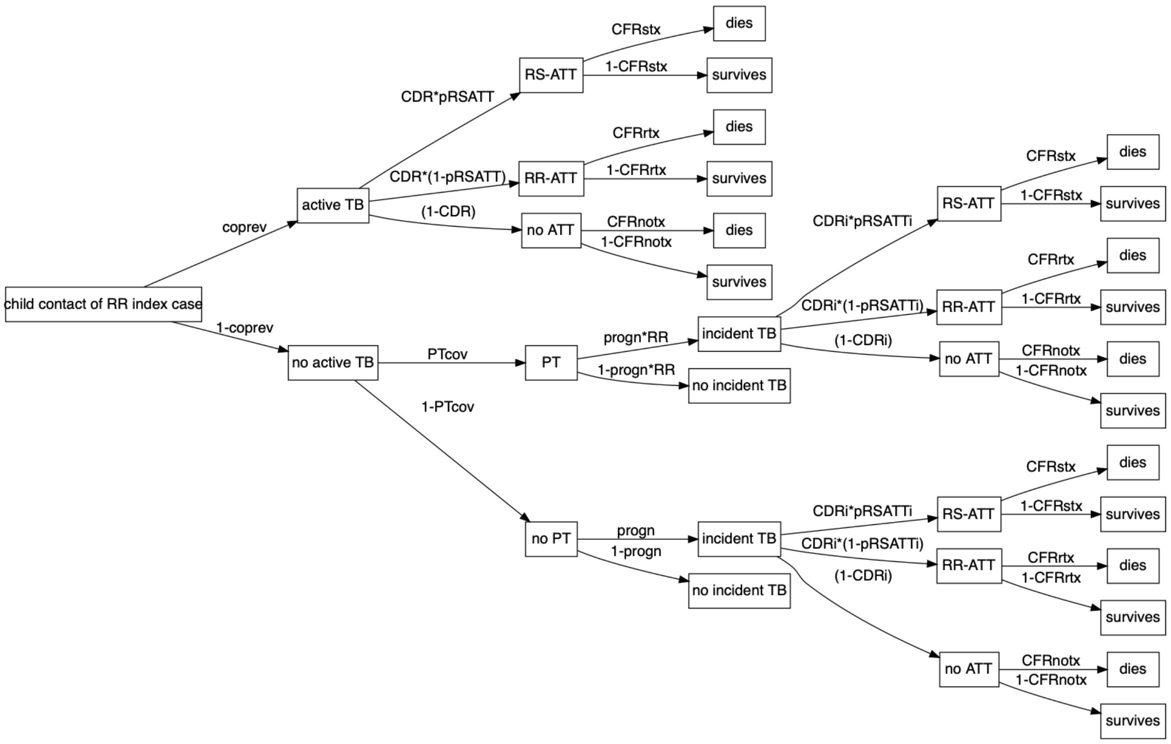

Figure 4 Decision tree model structure

Modelling approach & uncertainty quantification

The modelling used a decision tree with structure shown in Figure 4. Units of population 'flowing' through the tree were stratified by combinations of 'attributes' (here: 2 age categories [<5 years & 5-14 years]; 3 drug resistance types [RS, MDR/RR but fluoroquinolone-susceptible, MDR/RR and fluoroquinolone-resistant]; 3 HIV states [HIV-ve, HIV+ve/ART-ve, HIV+ve/ART+ve]) in proportions determined by the epidemiological modelling of each country described above. Each label on the decision tree transitions represents a function whose values can depend on the attribute combinations, i.e. the flow through the tree depends on age, HIV-status etc. Each function depends on underlying parameters described in Table 1 as described above. All calculations were performed as probabilistic sensitivity analyses (PSA). For the 215 countries in the analysis, 1,000 samples from all of the parameter distributions, together with 1,000 samples from the country-level estimates of fluoroquinolone resistance and household contacts, were used to generate a dataset of inputs. Functions derived from the tree to calculate mean outcomes for each unit of input data were applied to this dataset. Means and 95% quantiles as uncertainty intervals were reported for outcomes. Modelling was done with the HEdtree R package (https://github.com/petedodd/HEdtree).

Metrics calculated

For each set of inputs, we calculated drivers of resource use such as courses of first-line and MDR/RR-TB treatment, preventive therapy courses, and outcomes such as tuberculosis prevalence, incidence (stratified by drug-resistance status), and death (both in total, and resulting from incident and coprevalent tuberculosis separately). Deaths were used to calculate life-years lost for each intervention scenario (equivalent to DALYs given our neglect of reduced health related quality of life during tuberculosis disease), discounted by 3% per annum. The discly R package (https://github.com/petedodd/discly, whose data is from an analysis described in Dodd and colleagues[18]) was used to generate discounted life-expectancies for each country in 2020 at each age, and means were taken across our age categories assuming a uniform distribution of ages. Activity metrics were combined with unit cost predictions in each country to calculate intervention costs as described below.

Reproducibility

This analysis was run using R version 4.1.0. All code and data are available with guidance for use on GitHub at: https://github.com/petedodd/DRHHCM

Supplementary methods: costing

Resource use

We estimated the costs of resources used for household visits for screening, LTBI testing, tuberculosis testing, treatment of tuberculosis disease, and provision of TPT from a health system perspective. Resources used for tuberculosis household contact management (HHCM) intervention activities and provision of TPT in eligible contacts, included a single home visit for tuberculosis symptomatic screening, an HIV test for non-symptomatic children aged 5-14 years, a TST for non-symptomatic children aged 5-14 years and 6 months of daily TPT provision. Resource use for TPT included the drugs (either a fluoroquinolone [moxifloxacin or levofloxacin], bedaquiline or delamanid), monthly health facility follow-up visits, monthly laboratory monitoring (complete blood count and liver function tests) and management of adverse events experienced during TPT. The total doses for the drugs were estimated using weight band based dosing using the median weight for children in the age groups 0-4 and 5-14 years. We assumed the management of mild adverse events to consist of a single outpatient visit and laboratory testing (complete blood count and liver function tests) while serious adverse events would require 7 days hospitalisation and laboratory testing, similarly to Jo and colleagues.[19] Symptomatic contacts would require additional resources to confirm or rule out tuberculosis disease. We assumed symptomatic child contacts would require a single outpatient facility visit, a chest X-ray and an Xpert MTB/RIF test on a single sputum sample. We assumed young children in the 0-4 years age group would not be able to produce sputum necessitating collection of an induced sputum sample. Child contacts diagnosed with tuberculosis disease would be initiated on anti-tuberculosis treatment and incur resources as described below. A summary of tuberculosis HHCM intervention activities and associated resource utilisation for the different intervention scenarios is shown in Table 2.

The treatment of tuberculosis disease is based on the drug susceptibility test pattern of the strain and follows the current WHO guidance on the management of tuberculosis in children and adolescents.[20] In general, tuberculosis treatment requires the use of inpatient and outpatient care in addition to paediatric-specific anti-tuberculosis drugs. Rifampicin-susceptible tuberculosis (RS-TB) treatment requires the standard 6-month regimen (2 months HRZE + 4 months HR). We assumed the following all-oral anti-tuberculosis drug regimens for children with MDR/RR-TB depending on fluoroquinolone resistance profile: children <5 years with fluoroquinolone-resistant MDR/RR-TB: 12 months Bdq-Lzd-Cfz-Cs; children <5 years with fluoroquinolone-susceptible MDR/RR-TB: 12 months Bdq-Lfx-Lzd-Cfz; children 5-15 years with fluoroquinolone-resistant MDR/RR-TB: 15 months Bdq-Lzd-Cs-Cfz; and children 5-15 years with fluoroquinolone-susceptible MDR/RR-TB: 15 months Bdq-Lzd-Lfx-Cfz. These regimens were designed in line with the current guidance suggested by WHO and the Sentinel Project for Pediatric Drug-Resistant Tuberculosis. Resource use for inpatient and outpatient care (country and strain specific) was estimated using the average number of days spent in hospital, and the average number of outpatient visits to a health facility using data on utilisation of health services for tuberculosis reported by countries to WHO.[21] To avoid double counting, we assumed laboratory monitoring during treatment was included under National TB Programme (NTP) programme costs (see details on costs) and treatment for adverse events would be included as part of inpatient and outpatient care. A summary of the resources used for the treatment of tuberculosis disease is provided in Table 3.

Table 2 Summary of tuberculosis household contact management intervention activities and associated resource utilization. TPT=tuberculosis preventive therapy, <5 & HIV=children younger than five and those younger than 15 living with HIV/AIDS, <5 & HIV & TST=children younger than five and those younger than 15 who are living with HIV/AIDS or are tuberculin skin test positive, <15=all children younger than 15, Fq=fluoroquinolone (levofloxacin or moxifloxacin), Bdq/Dlm=bedaquilin or delamanid. Shading indicates where an activity requires a certain resource. Note that drug costs, monitoring and adverse events vary depending on the regimen used.

Intervention								
Household contact management	No	Yes						
TPT recipients	None	None	<5 & HIV		<5 & HIV & TST		<15	
TPT regimen	None	None	Fq	Bdq/Dlm	Fq	Bdq/Dlm	Fq	Bdq/Dlm
Household contact management activities								
Home visit								
HIV test for children 5-15								
TST for children 5-15								
Tuberculosis testing if contact found symptomatic								
Outpatient department visit								
Chest X-ray								
Xpert MTB/RIF								
TPT								
Drug costs								
Follow up								

Monitoring									
Adverse events									

Table 3 Summary of tuberculosis testing and treatment resource utilisation, independent of intervention. MDR/RR-TB=multidrug- or rifampicin-resistant tuberculosis, RS-TB=drug-susceptible tuberculosis, Lzd=Linezolind, Cs=Cycloserine, Cfz=Clofazimine, Dlm=Delaminid, Bdq=bedaquiline, Lfx=Levofloxacin, H=isoniazid, R=rifampicin, Z=Pyrazinamide, E=ethambutol.

Activity	Resources	Resource description
MDR/RR-TB treatment	HIV test	Health facility-based HIV testing per testing episode/person.
	Inpatient care	Inpatient care (hospitalisation) for the entire MDR/RR-TB episode per patient.
	Outpatient care	Outpatient care (health facility treatment follow-up visits) for the entire MDR/RR-TB episode per patient.
	NTP programme costs	NTP programme costs per patient starting MDR-TB/XDR-TB treatment.
	Drugs	Children <5 years with fluoroquinolone resistant MDR/RR-TB: 12 months Bdq-Lzd-Cfz-Cs
		Children <5 years with fluoroquinolone susceptible MDR/RR-TB: 12 months Bdq-Lfx-Lzd-Cfz
		Children 5-15 years with fluoroquinolone resistant MDR/RR-TB: 15 months Bdq-Lzd-Cs-Cfz
		Children 5-15 years with fluoroquinolone susceptible MDR/RR-TB: 15 months Bdq-Lzd-Lfx-Cfz
	Laboratory monitoring	Children < 5years: full blood count and electrocardiogram every 2 months for 12 months
		Children 5-15 years: full blood count and electrocardiogram every 2 months for 15 months
RS-TB treatment	HIV test	Health facility-based HIV testing per testing episode/person.
	Inpatient care	Inpatient care (hospitalisation) for the entire DS-TB episode per patient.
	Outpatient care	Outpatient care (health facility treatment follow-up visits) for the entire DS-TB episode per patient.
	NTP programme costs	NTP programme costs per patient starting first-line tuberculosis treatment.
	Drugs	2 months H-R-Z-E + 4 months H-R
	Laboratory monitoring	Assumed no laboratory monitoring during treatment

Unit costs

Sources and assumptions

The costs for all the resources used in the different intervention scenarios were estimated by attaching monetary values using relevant unit costs estimated for each country. All unit costs used in this analyses were estimated using publicly available data or published literature (see Table 4). We estimated all costs from the healthcare provider's perspective in 2020 USD prices. All costs were

estimated as means and standard deviations to quantify uncertainty. For studies reporting the range (or 95% uncertainty range), the standard deviation was estimated as approximately equal to the range of the data divided by 4. We assumed +/- 50% relative uncertainty for cost parameters reported without SDs or uncertainty ranges. We estimated uncertainty for the costs resulting from adding individual unit costs as the square root of the sum of squares of the individual standard deviations. All historical costs were adjusted for inflation to 2020 prices using relevant gross domestic product (GDP) deflators available from the World Bank[22]. Costs expressed in US$ were first converted back to the local currency using the official exchange rate (local currency unit per US$) relating to the time period the cost data were collected. The costs were then inflated using GDP price deflators and converted back to US$ using the official exchange rate for 2020. All costs derived from a specific country were transferred to other countries by applying relevant purchasing power parity conversion factors available from the World Bank[22], to adjust for different price levels. Costs were assumed to accrue in the present, with no discounting applied. Unit costs for each country were modelled as following gamma distributions with means/SDs corresponding to estimates.

Table 4 Unit costs of tuberculosis screening, tuberculosis testing and diagnosis, tuberculosis treatment and LTBI treatment.

**Resource**	**Cost parameter**	**Description**	**Unit cost ($US)**	**Unit cost parameter estimation assumptions**
Household visit	c_hh_visit	Household visit to perform contact tracing and screening for prevalent tuberculosis	3.36 (0.8)	Estimated based on costs for active case finding per household contact reported in a study from Peru (Shah et al.[23])
HIV test	c_hiv_test	Facility-based HIV testing	Income-group specific	Estimated based on income level group (low- to upper-middle income countries versus high income countries) specific health facility-based HIV testing costs per testing episode from Johnson et al.[24]
Tuberculin skin test	c_tst_test	Tuberculin skin test	7.26 (1.9)	Estimated based on costs for a tuberculin skin test with purified protein derivative (PPD RT 23) reported in a recent study from Brazil (Steffen et al.[25])
Outpatient visit	c_opd_visit	Outpatient visit to a primary-level hospital in the public sector	Country-specific	Estimated using a WHO CHOosing Interventions that are Cost-Effective (WHO-CHOICE) based econometric model using the number of total outpatient visits per facility per year, visits per provider per day and GDP per capita as predictors.[26]
Chest X-ray	c_cxr_exam	Chest radiograph or chest X-ray	4.04 (1.1)	Estimated based on costs for a chest radiograph reported in a recent study from Brazil (Steffen et al.[25])
Xpert MTB/RIF test (0-4	c_xpert_test.04	GeneXpert MTB/RIF molecular test in	24.1 (2.1)	Comprises of the costs for collecting an induced sputum sample (assuming inability to spontaneously produce sputum

years)		children below 5 years		in young child) based on estimates provided by the Paediatric Operational Sustainability Expertise Exchange group (POSEE group)[27] and the costs for a Xpert MTB/RIF test on a single sample reported in a multi-laboratory study from India (Sarin et al.[28]).
Xpert MTB/RIF test (5-14 years)	c_xpert_test.514	GeneXpert MTB/RIF molecular test in children over 5 years	17.5 (0.8)	Comprises the costs for collecting self-expectorated sputum based on recent estimates from Uganda (Tucker et al.[29]) and the costs for a Xpert MTB/RIF test on a single sample reported in a multi-laboratory study from India (Sarin et al.[28]).
Isoniazid-based TPT (0-4 years)	c_tpt_INH.04	Isoniazid-based TPT in children below 5 years	5.4 (1.4)	Estimated using weight band based dosing using the median weight for children in the 0-4 years age group and applying unit costs available from the Global Drug Facility for a 6 month treatment on isoniazid-based TPT.[30]
Isoniazid-based TPT (5-14 years)	c_tpt_INH.514	Isoniazid-based TPT in children over 5 years	12.6 (2.3)	Estimated using weight band based dosing using the median weight for children in the 5-14 years age group and applying unit costs available from the Global Drug Facility for a 6 month treatment on isoniazid-based TPT.[30]
Levofloxacin-based TPT (0-4 years)	c_tpt_LVX.04	Levofloxacin-based TPT in children below 5 years	34.2 (11.7)	Estimated using weight band based dosing using the median weight for children in the 0-4 years age group and applying unit costs available from the Global Drug Facility for a 6 month treatment on levofloxacin-based TPT.[30]
Levofloxacin-based TPT (5-14 years)	c_tpt_LVX.514	Levofloxacin-based TPT in children over 5 years	46.8 (22.9)	Estimated using weight band based dosing using the median weight for children in the 5-14 years age group and applying unit costs available from the Global Drug Facility for a 6 month treatment on levofloxacin-based TPT.[30]
Moxifloxacin-based TPT (0-4 years)	c_tpt_MXF.04	Moxifloxacin-based TPT in children below 5 years	54 (9.0)	Estimated using weight band based dosing using the median weight for children in the 0-4 years age group and applying unit costs available from the Global Drug Facility for a 6 month treatment on moxifloxacin-based TPT.[30]
Moxifloxacin-based TPT (5-14 years)	c_tpt_MXF.514	Moxifloxacin-based TPT in children below 5 years	144 (9.0)	Estimated using weight band based dosing using the median weight for children in the 5-14 years age group and applying unit costs available from the Global Drug Facility for a 6 month treatment on

				moxifloxacin-based TPT.[30]
Bedaquiline-based TPT (0-4 years)	c_tpt_BDQ.04	Bedaquiline-based TPT n children below 5 years	74.18 (24.1)	Estimated using weight band based dosing using the median weight for children in the 5-14 years age group and applying unit costs available from the Global Drug Facility for a 6 month treatment on bedaquiline-based TPT.[30]
Bedaquiline-based TPT (5-14 years)	c_tpt_BDQ.514	Bedaquiline-based TPT in children over 5 years	160.28 (97.1)	Estimated using weight band based dosing using the median weight for children in the 5-14 years age group and applying unit costs available from the Global Drug Facility for a 6 month treatment on bedaquiline-based TPT.[30]
Delamanid-based TPT (0-4 years)	c_tpt_DLM.04	Delamanid-based TPT in children below 5 years	203.4 (25.7)	Estimated using weight band based dosing using the median weight for children in the 5-14 years age group and applying unit costs available from the Global Drug Facility for a 6 month treatment on delamanid-based TPT.[30]
Delamanid-based TPT (5-14 years)	c_tpt_DLM.514	Delamanid-based TPT in children over 5 years	406.8 (127.4)	Estimated using weight band based dosing using the median weight for children in the 5-14 years age group and applying unit costs available from the Global Drug Facility for a 6 month treatment on delamanid-based TPT.[30]
TPT follow-up	c_tpt_fu	TPT follow-up	Country-specific	Consists of monthly outpatient health facility visits for the duration of TPT, estimated using a WHO CHOosing Interventions that are Cost-Effective (WHO-CHOICE) based econometric model using the number of total outpatient visits per facility per year, visits per provider per day and GDP per capita as predictors.[26]
Isoniazid-based TPT monitoring	c_monit_INH	Isoniazid-based TPT monitoring	Country-specific	This TPT treatment monitoring cost comprises of the cost of performing monthly liver function tests (LFTs), estimated based on costs for LFTs reported in a study from Brazil (Steffen et al.[25]).
Fluoroquinolone-based TPT monitoring	c_monit_FQ	Fluoroquinolone-based TPT monitoring	Country-specific	
Bedaquiline-based TPT monitoring	c_monit_BDQ	Bedaquiline-based TPT monitoring	Country-specific	
Isoniazid-based TPT mild adverse events	c_aes_INH	Isoniazid-based TPT mild adverse events treatment	Country-specific	The cost of treating children experiencing mild adverse events while receiving TPT and comprises one-time outpatient visit and laboratory testing (complete blood

treatment				count, electrolyte panel, urinalysis and liver function tests). The outpatient visit cost was estimated using a WHO-CHOICE based econometric model as described before.[31] Laboratory testing costs were estimated based on costs for LFTs and CBC reported in a study from Brazil (Steffen et al.[25]).
Fluoroquinolone-based TPT mild adverse events treatment	c_aes_FQ	Fluoroquinolone-based TPT mild adverse events treatment	Country-specific	
Bedaquiline-based TPT mild adverse events treatment	c_aes_BDQ	Bedaquiline-based TPT mild adverse events treatment	Country-specific	
Isoniazid-based TPT serious adverse events treatment	c_saes_INH	Isoniazid-based TPT serious adverse events treatment	Country-specific	The cost of treating children experiencing serious adverse events while receiving TPT and comprises 7 day hospitalisation and laboratory testing (complete blood count, electrolyte panel, urinalysis and liver function tests). The inpatient cost was estimated using a WHO-CHOICE based econometric model as described before.[26] Laboratory testing costs were estimated based on costs for LFTs and CBC reported in a study from Brazil (Steffen et al.[25]).

Estimation of tuberculosis disease treatment costs

We estimated tuberculosis disease treatment costs using publicly available data based on an approach described in the WHO Global Tuberculosis Report 2019[32] as the sum of NTP expenditures, costs for inpatient and outpatient care and the cost of paediatric specific anti-tuberculosis drugs.

NTP expenditures are based on the sum of the national expenditures reported by country NTPs, available from the WHO global Tuberculosis database[21] and as described in the WHO World Tuberculosis Reports.[33] Categories of expenditure included in the data include 1) laboratory infrastructure, equipment and supplies; 2) NTP staff at central and subnational levels (e.g. NTP managers, and provincial or district tuberculosis coordinators); 3) programme costs (for example, management and supervision, training, policy development, meetings); 4) operational research, including surveys; and, 5) patient support activities; 6) First-line and second-line drugs; 7) Programme costs specifically related to MDR/RR-TB; and 8) Collaborative tuberculosis/HIV activities. In our analysis we excluded expenditures on drugs used to treat RS-TB (first-line drugs) and drug-resistant tuberculosis (second-line drugs) to allow for inclusion of paediatric-specific anti-tuberculosis drugs separately.

Outpatient care costs were estimated by multiplying the number of visits to a health facility for patients starting tuberculosis treatment (first-line for RS-TB or second-line for MDR/RR-TB) by

the unit cost for an outpatient visit to a primary-level hospital in the public sector estimated using a WHO CHOosing Interventions that are Cost-Effective (WHO-CHOICE) based econometric model using facility characteristics (ownership - public or private, location - urban or rural and level), number of outpatient visits per facility per year, number of visits per provider per day and GDP per capita as explanatory variables.[26]

Inpatient care costs were estimated using the percentage of patients starting tuberculosis treatment (first-line for RS-TB or second-line for MDR/RR-TB) that are hospitalised, the average duration of stay (days), and the unit cost for inpatient care in a primary-level hospital in the public sector estimated using a WHO-CHOICE based econometric model using facility characteristics (ownership - public or private and level), bed occupancy rate, average length of stay, number of inpatient admissions and GDP per capita as explanatory variables.[26]

Country-specific missing data on outpatient or inpatient care utilisation (n=60 countries) was imputed using regional averages. Missing costs for NTP programme management costs (n=130 countries) were estimated using a cost regression model based on countries with data (fitted using countries with data and the latest GDP data available from the World Bank[34]). See Figure 5 for regression of NTP costs used to predict missing country values, and Figure 6 for the regression analysis used for missing inpatient and outpatient cost prediction.

The costs of anti-tuberculosis drugs and pyridoxine were estimated using weight band based dosing and applying unit costs available from the Global Drug Facility.[35]

Figure 5 Regression of NTP costs against per capita GDP

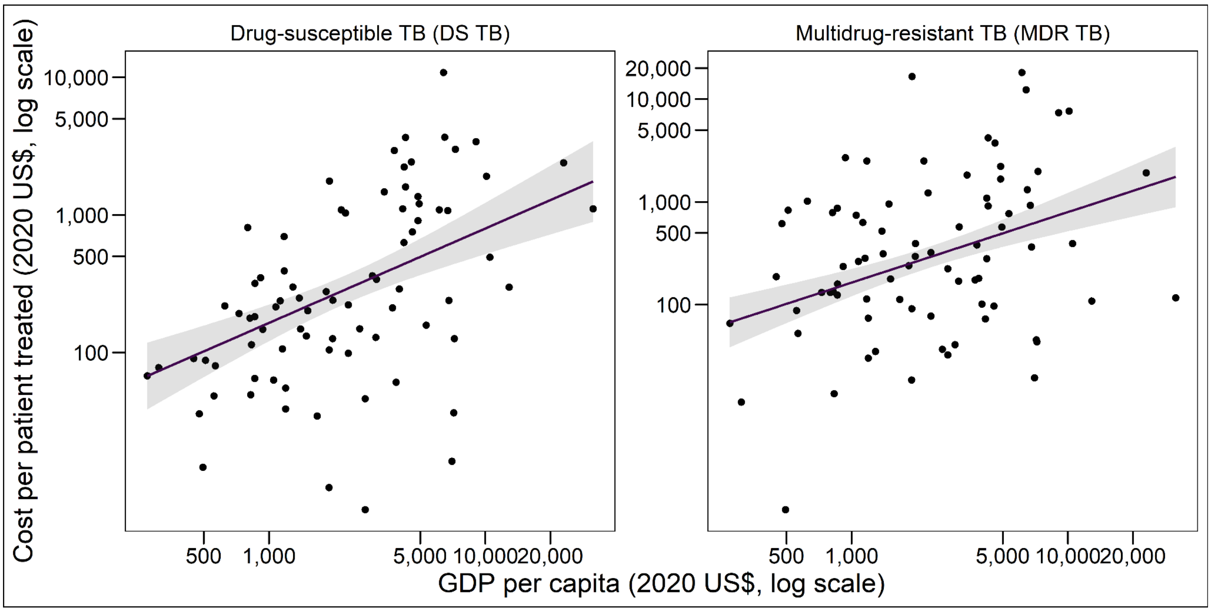

Figure 6 Regression of inpatient and outpatient treatment costs against GDP per capita

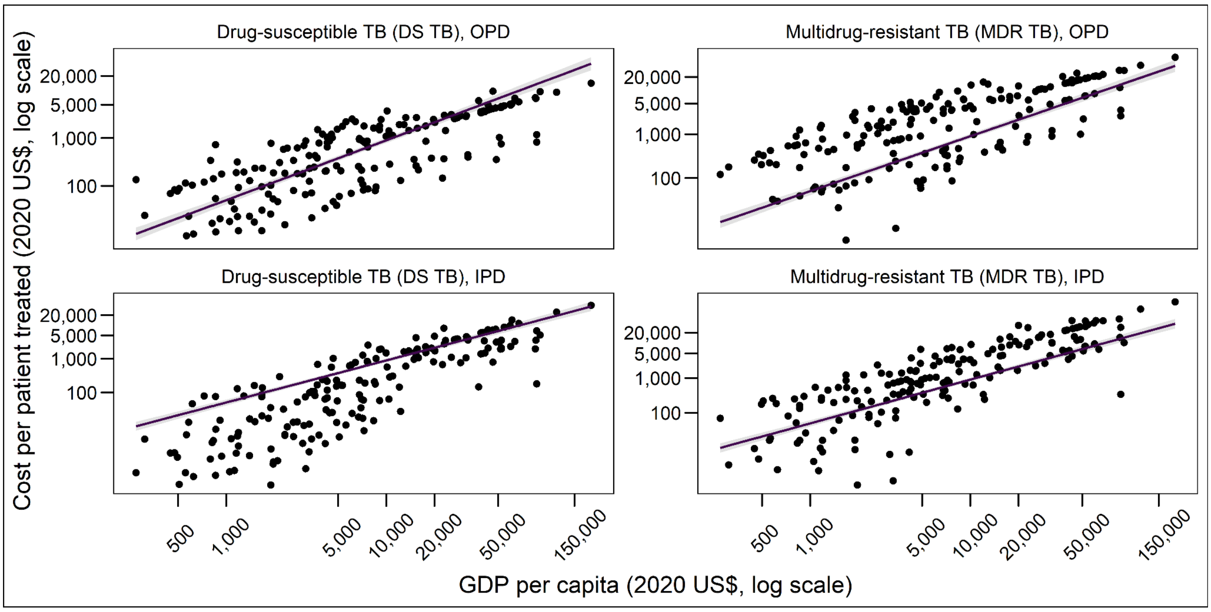

Estimates of implied cost-effectiveness thresholds and GDP per capita

Historically, where explicit cost-effectiveness thresholds are lacking for countries, 1-3 x GDP was used as a guide. More recent work has suggested this is too high as an estimate of marginal ICERs in current health systems. Ochalek and colleagues[36] and Woods and colleagues[37] have used different methods to estimate cost-effectiveness thresholds for large numbers of countries, shown in relation to each other and 0.5 x GDP and 1 x GDP in Figure 7. Ochalek and colleagues' estimates are based on country-data whereas Woods and colleagues' extrapolate from a UK estimate. Both sets of estimates carry substantial uncertainty; those of Ochalek and colleagues are typically higher than those of Woods and colleagues. Based on these data, we use 0.5 x GDP as a contextual guide, as noted in Chi and colleagues.[38] The choice of threshold is ultimately for decision makers.

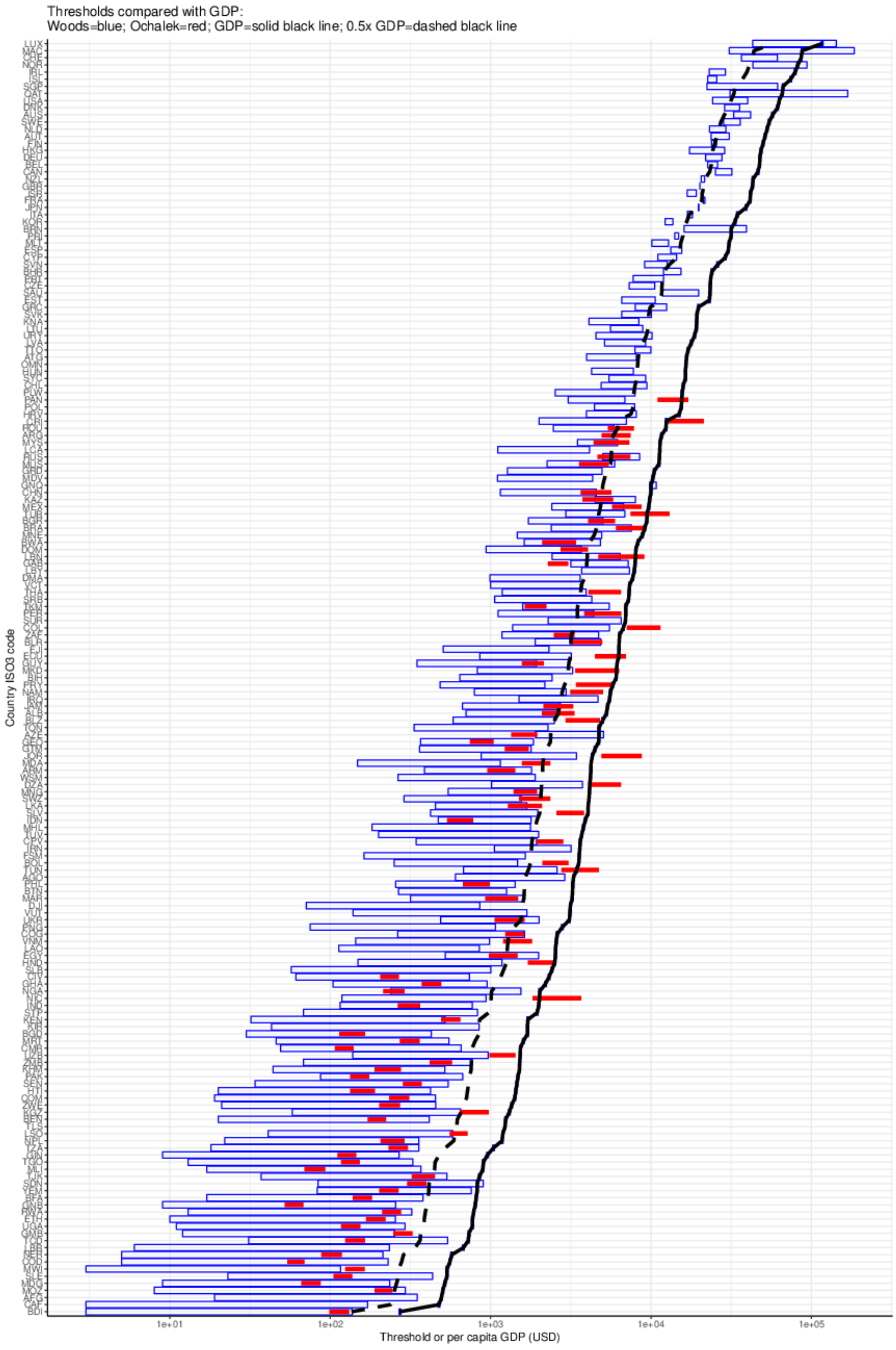

Figure 7 Comparison between econometric estimates of implied cost-effectiveness thresholds based on marginal health spending and per capita GDP

Supplementary results

Resources per case or death averted

Figure 8 shows the resources needed (in terms of children screened or TPT courses) per incident tuberculosis case or death averted.

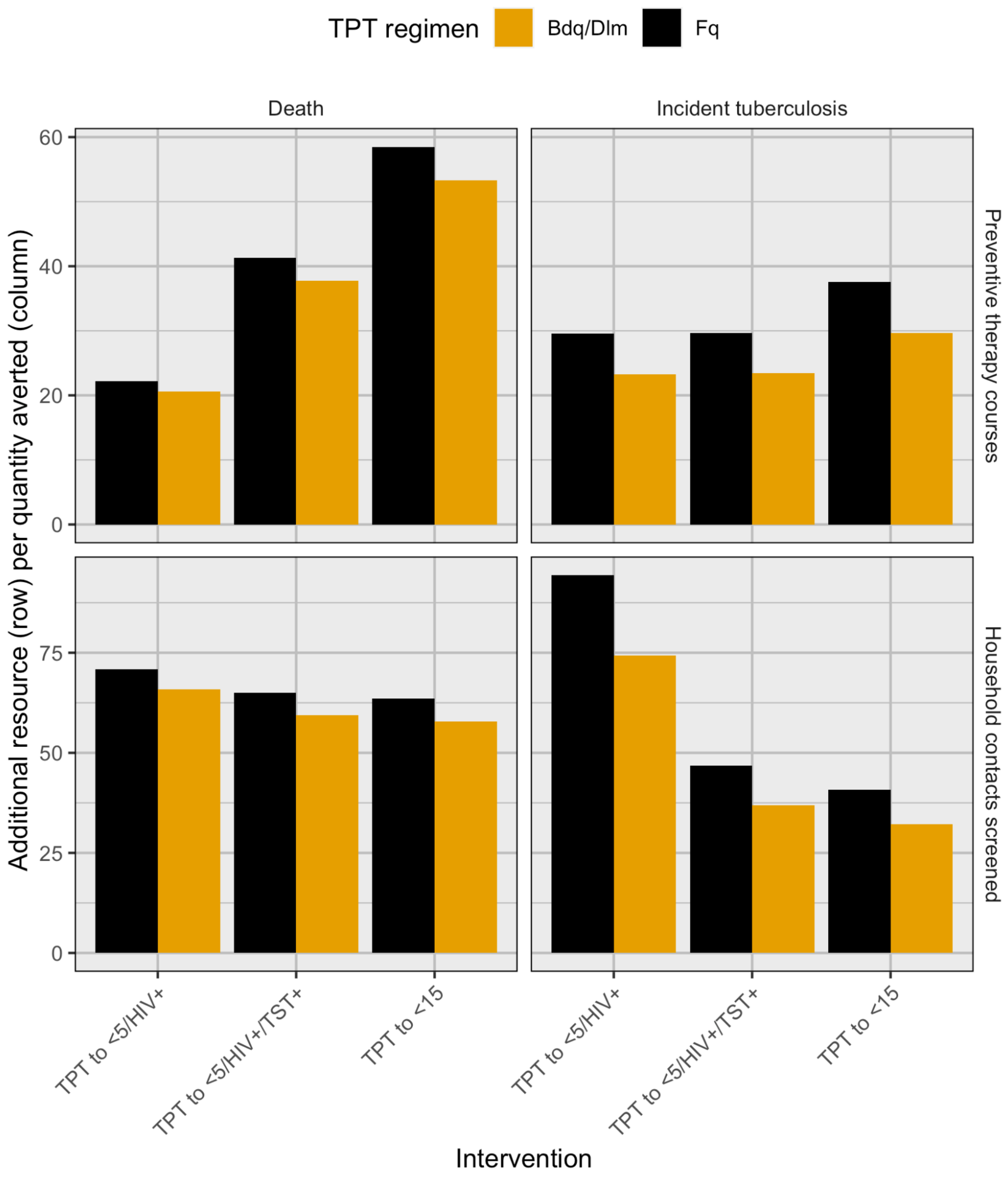

Figure 8 Impact of household contact management in children younger than 15 as incremental demands on the health system (preventive therapy courses [row 1] or household contacts screened [row 2]) required to avert one tuberculosis death (column 1) or tuberculosis case (column 2).
TPT=tuberculosis preventive therapy, <5 & HIV=children younger than five and those younger than 15 living with HIV, <5 & HIV & TST=children younger than five and those younger than 15 who are living with HIV or are tuberculin skin test positive, <15=all children younger than 15, Fq=fluoroquinolone (levofloxacin or moxifloxacin), Bdq/Dlm=bedaquilin or delamanid.

Comparison to previous work

Since our previous work,[1] a large individual-patient meta-analysis of progression risks in child close contacts has been published (Martinez and colleagues[7]), so we updated the risks of progression to incident tuberculosis to make use of these data. We also used the estimates in Martinez and colleagues[7] for the efficacy of TPT in preventing incident tuberculosis, rather than the review of Ayieko and colleagues.[10] For this analysis we based co-prevalence on a systematic review and meta-analysis specific to drug-resistant tuberculosis,[6] rather than the review of Fox and colleagues we used previously.[5]

Because of these changes, and in order to compare the resource needs per outcome with those reported in the main text for MDR/RR-TB, we re-ran our model with assumptions that corresponded to all tuberculosis being RS-TB. Figure 9 shows the equivalent of Figure 8 above, and reports the number of TPT courses and child contacts screened to avert one incident case or one death. As noted in the main article, fewer children need to be screened to avert a death in the case of MDR/RR-TB than RS-TB; TPT courses to avert an incident case depend on regimen for MDR/RR-TB but are comparable to RS-TB. Comparing with our previous estimates for HHCM with TPT to <5/HIV+/TST+, our revised parametrization results in 85 vs 77 children screened to prevent a death. Comparing with our previous estimates for HHCM with TPT to <5/HIV+/TST+, our revised parametrization results in 24 vs 42 TPT courses to prevent a case. These differences are likely driven by the higher efficacy of TPT assumed (RR=0.37 vs RR=0.65).

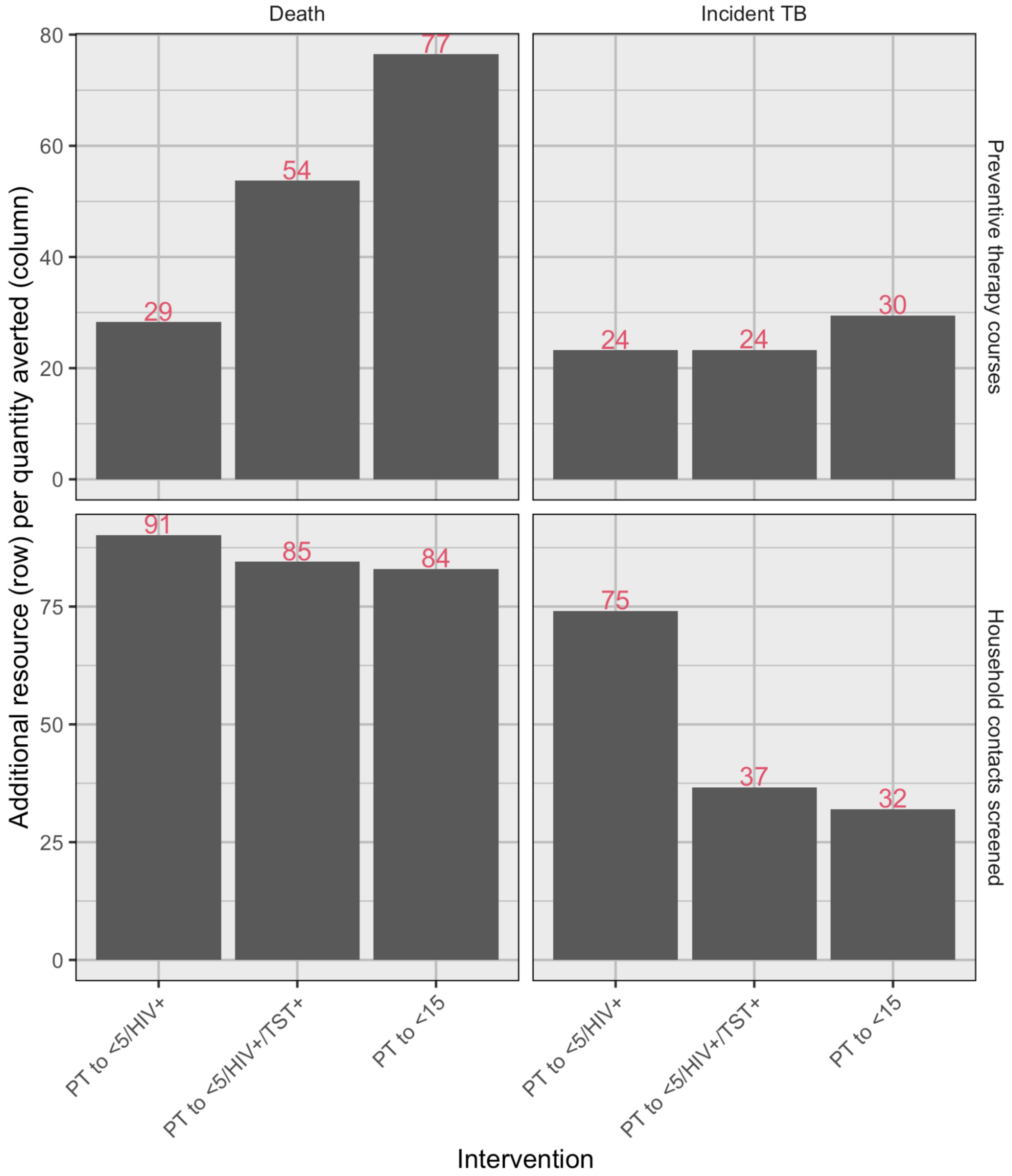

Figure 9 Number of preventive therapy courses and household contacts screened per incident tuberculosis case and tuberculosis deaths averted assuming all tuberculosis (and tuberculosis treatment) is RS-TB

Additional health economic outputs

In the main text, Figure 3 shows the incremental cost-effectiveness ratios (ICERs) for the WHO high MDR/RR-TB burden countries; Figure 10 below shows the same plot including all countries in the analysis with all the relevant data. Figure 10 includes 1x, 0.5x and, additionally, 0.2x per capita GDP lines as proxy reference points for cost-effectiveness thresholds (see discussion above). Including

uncertainty in ICER estimates can be problematic; the cost-effectiveness acceptability curve or CEAC (Figure 11) provides an approach to representing uncertainty by plotting the probability an intervention is cost-effective at various thresholds (x-axis).

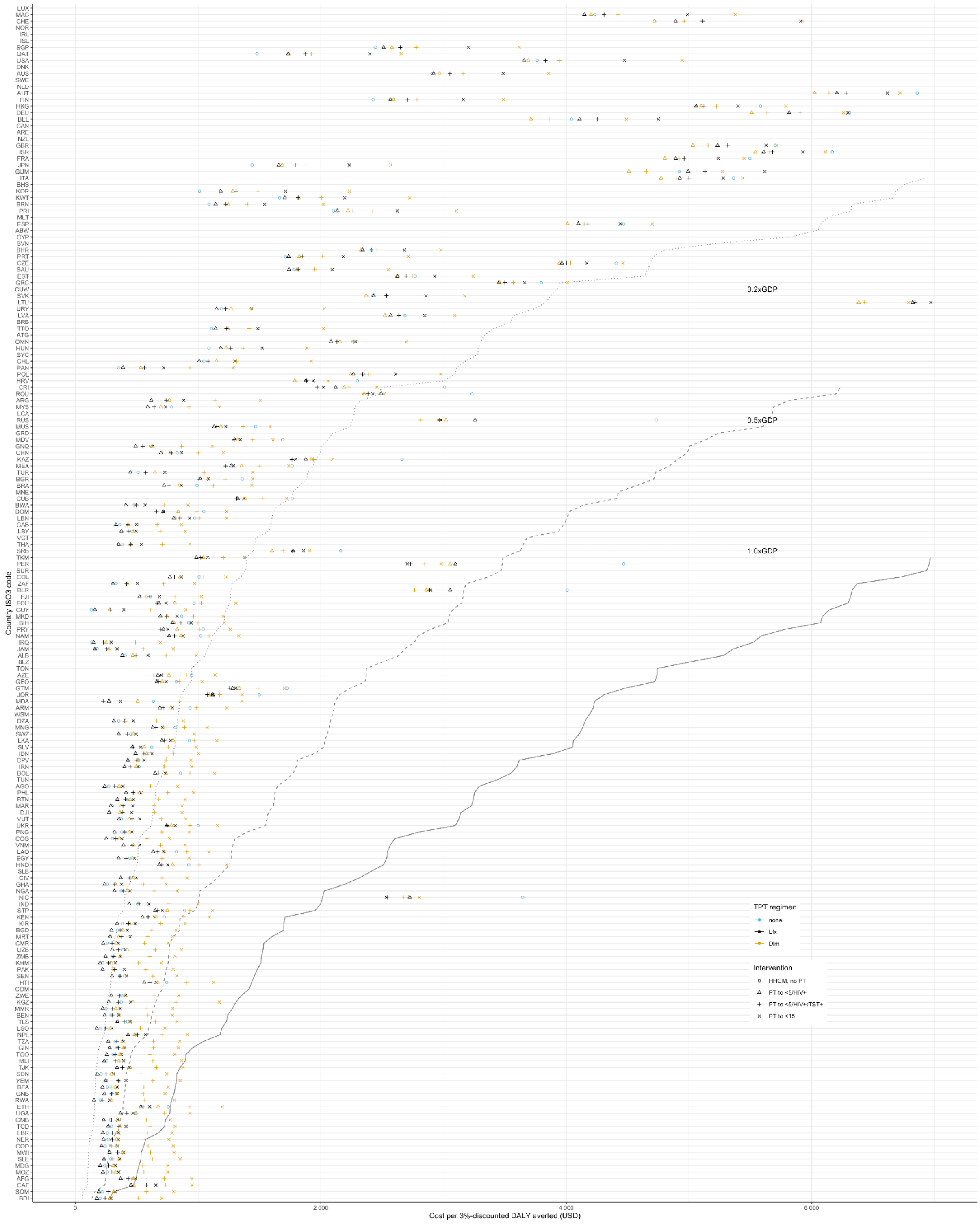

Figure 10 Incremental cost-effectiveness ratios (ICERs) of different interventions and TPT regimens by country (points), with lines showing per capita GDP and multiples of this for reference. ICER estimates are available on the GitHub repository here.

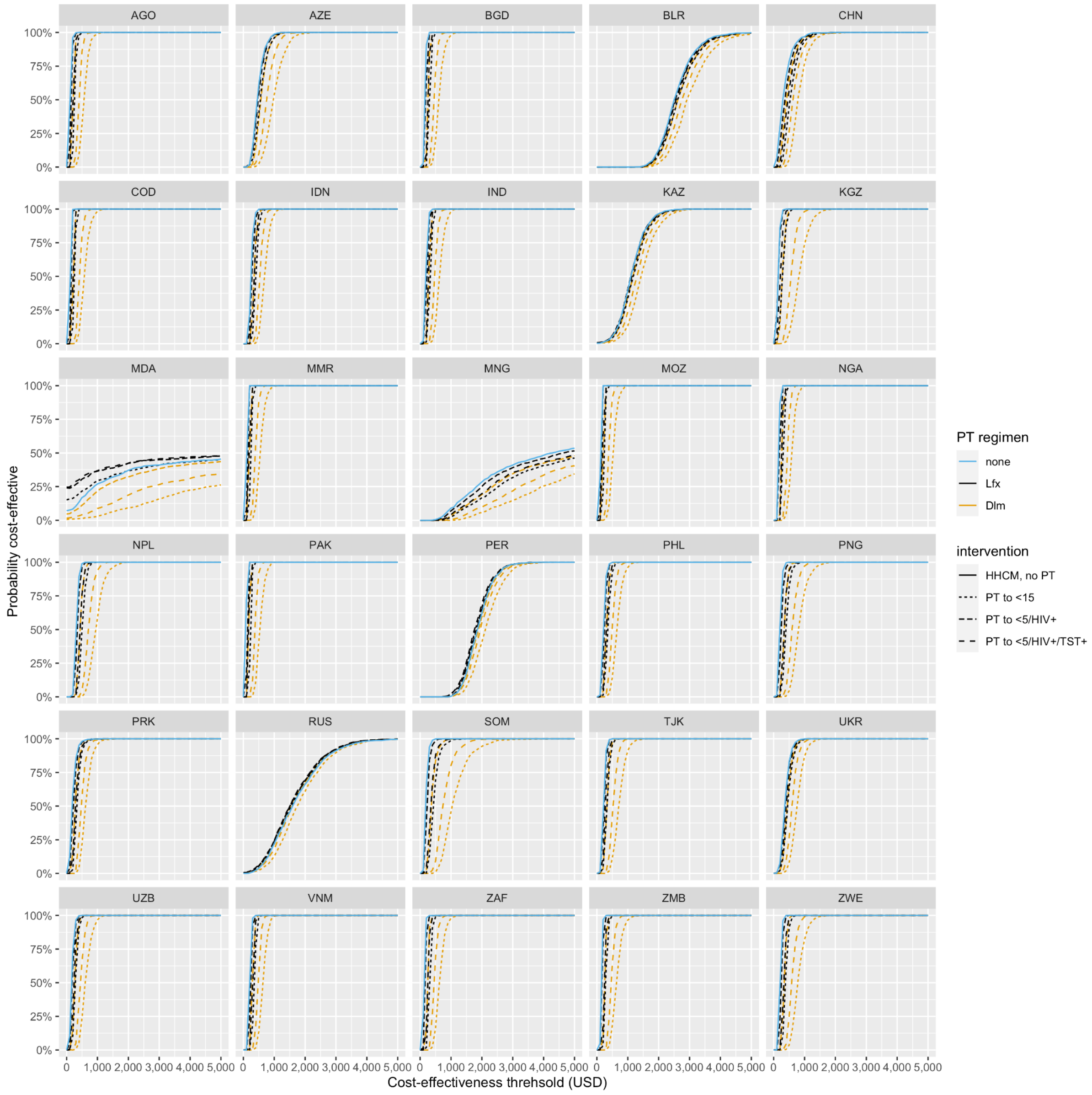

Figure 11 Cost-effectiveness acceptability curves for the 30 high MDR/RR-TB burden countries

Sensitivity analysis around the fraction of MDR/RR-TB that is pulmonary

As described above, our main analysis assumed that all notified adult MDR/RR-TB patients have pulmonary tuberculosis, making them eligible to trigger household contact management. This was based on the judgement that most MDR/RR-TB patients would have been diagnosed as such on the basis of bacteriological tests, most likely on pulmonary samples. Our previous analysis for all tuberculosis used older country data on disease type to model only a fraction of adult notifications were pulmonary. As a sensitivity analysis, we applied our previous method here.

For this, the proportion of notifications for each country that was pulmonary was based on aggregated country data, and regional averages where country data were lacking. The age- and sex-specific pulmonary notification estimates for each country were combined with age- and sex-specific predictions of 0-4 and (separately) 5-14 year old household contact numbers to estimate the average number of child household contacts of each age category per tuberculosis patient in each country

The results of this analysis are shown below in Table 5 (resources required; the analogue of the top half of Table 1 in main article); Table 6 (outcomes; the analogue of the bottom half of Table 1 in main article); and Table 7 (health economics; the analogue of Table 2 in main article). Results depending on absolute numbers were approximately 15% lower.

Table 5 Resource use for assumption that not all MDR/RR-TB index patients have pulmonary disease

	Intervention							
Household contact management	No	Yes						
TPT recipients	None	None	<5 & HIV		<5 & HIV & TST		<15	
TPT regimen	None	None	Fq	Bdq/Dlm	Fq	Bdq/Dlm	Fq	Bdq/Dlm
**Total resources**								
Households contacts screened	0 (0 - 0)	194,000 (174,000 - 217,000)	194,000 (174,000 - 217,000)	194,000 (174,000 - 217,000)	194,000 (174,000 - 217,000)	194,000 (174,000 - 217,000)	194,000 (174,000 - 217,000)	194,000 (174,000 - 217,000)
TPT courses	0 (0 - 0)	0 (0 - 0)	60,900 (53,900 - 68,400)	60,900 (53,900 - 68,400)	123,000 (110,000 - 138,000)	123,000 (110,000 - 138,000)	178,000 (160,000 - 200,000)	178,000 (160,000 - 200,000)
RS-TB treatments	10,800 (8,690 - 13,100)	6,790 (5,410 - 8,390)	5,990 (4,740 - 7,520)	5,840 (4,590 - 7,320)	4,930 (3,920 - 6,030)	4,560 (3,640 - 5,570)	4,620 (3,700 - 5,650)	4,190 (3,360 - 5,110)
MDR/RR-TB treatments	4,370 (3,000 - 6,070)	14,300 (12,400 - 16,500)	14,000 (12,200 - 16,300)	13,900 (12,100 - 16,200)	13,700 (11,800 - 15,900)	13,400 (11,700 - 15,400)	13,600 (11,800 - 15,700)	13,200 (11,600 - 15,200)
**Incremental resources**								
Households contacts screened		194,000 (174,000 - 217,000)	194,000 (174,000 - 217,000)	194,000 (174,000 - 217,000)	194,000 (174,000 - 217,000)	194,000 (174,000 - 217,000)	194,000 (174,000 - 217,000)	194,000 (174,000 - 217,000)
TPT courses		0 (0 - 0)	60,900 (53,900 - 68,400)	60,900 (53,900 - 68,400)	123,000 (110,000 - 138,000)	123,000 (110,000 - 138,000)	178,000 (160,000 - 200,000)	178,000 (160,000 - 200,000)
RS-TB treatments		-4,000 (-5,720 - -2,480)	-4,790 (-6,490 - -3,230)	-4,940 (-6,650 - -3,400)	-5,850 (-7,760 - -4,230)	-6,220 (-8,190 - -4,600)	-6,160 (-8,110 - -4,510)	-6,590 (-8,590 - -4,930)
MDR/RR-TB treatments		9,900 (8,000 - 11,800)	9,660 (7,790 - 11,600)	9,550 (7,680 - 11,500)	9,300 (7,390 - 11,300)	9,010 (7,070 - 10,900)	9,190 (7,200 - 11,200)	8,850 (6,900 - 10,800)

Table 6 Outcomes for assumption that not all MDR/RR-TB index patients have pulmonary disease

	Intervention							
Household contact management	No	Yes						
TPT recipients	None	None	<5 & HIV		<5 & HIV & TST		<15	
TPT regimen	None	None	Fq	Bdq/Dlm	Fq	Bdq/Dlm	Fq	Bdq/Dlm
**Total outcomes**								
Incident tuberculosis	9,660 (7,930 - 11,700)	9,660 (7,930 - 11,700)	7,570 (6,080 - 9,390)	7,020 (5,610 - 8,720)	5,450 (4,360 - 6,670)	4,350 (3,530 - 5,280)	4,840 (3,840 - 5,930)	3,570 (2,910 - 4,340)
Incident RS-TB	1,690 (1,210 - 2,340)	1,690 (1,210 - 2,340)	1,230 (856 - 1,780)	1,230 (856 - 1,780)	761 (541 - 1,060)	761 (541 - 1,060)	627 (450 - 889)	627 (450 - 889)
Incident MDR/RR-TB	7,970 (6,260 - 9,960)	7,970 (6,260 - 9,960)	6,340 (4,880 - 7,910)	5,790 (4,430 - 7,350)	4,690 (3,640 - 5,890)	3,590 (2,800 - 4,470)	4,210 (3,280 - 5,290)	2,950 (2,320 - 3,650)
Incident tuberculosis deaths	2,160 (1,720 - 2,670)	2,160 (1,720 - 2,670)	1,410 (1,120 - 1,740)	1,210 (959 - 1,500)	1,160 (921 - 1,460)	890 (715 - 1,110)	1,090 (862 - 1,370)	798 (637 - 992)
Prevalent tuberculosis deaths	3,060 (2,610 - 3,570)	1,050 (874 - 1,250)	1,050 (874 - 1,250)	1,050 (874 - 1,250)	1,050 (874 - 1,250)	1,050 (874 - 1,250)	1,050 (874 - 1,250)	1,050 (874 - 1,250)
**Incremental outcomes**								
Incident tuberculosis		0 (0 - 0)	-2,100 (-2,650 - -1,630)	-2,640 (-3,300 - -2,080)	-4,210 (-5,190 - -3,350)	-5,310 (-6,420 - -4,350)	-4,830 (-5,950 - -3,830)	-6,090 (-7,370 - -4,950)
Incident RS-TB		0 (0 - 0)	-463 (-676 - -318)	-463 (-676 - -318)	-931 (-1,310 - -665)	-931 (-1,310 - -665)	-1,070 (-1,480 - -767)	-1,070 (-1,480 - -767)
Incident MDR/RR-TB		0 (0 - 0)	-1,630 (-2,160 - -1,220)	-2,180 (-2,820 - -1,690)	-3,280 (-4,190 - -2,530)	-4,380 (-5,430 - -3,470)	-3,760 (-4,780 - -2,890)	-5,020 (-6,280 - -3,940)
Incident tuberculosis deaths		0 (0 - 0)	-748 (-978 - -563)	-950 (-1,230 - -732)	-995 (-1,260 - -770)	-1,270 (-1,570 - -1,020)	-1,070 (-1,340 - -830)	-1,360 (-1,690 - -1,090)
Prevalent tuberculosis deaths		-2,010 (-2,400 - -1,660)	-2,010 (-2,400 - -1,660)	-2,010 (-2,400 - -1,660)	-2,010 (-2,400 - -1,660)	-2,010 (-2,400 - -1,660)	-2,010 (-2,400 - -1,660)	-2,010 (-2,400 - -1,660)

Table 7 Health-economic outputs for assumption that not all MDR/RR-TB index patients have pulmonary disease

	**Intervention**													
Household contact management	No	Yes												
TPT recipients	None	None	<5 & HIV				<5 & HIV & TST				<15			
TPT regimen	None	None	Levofloxacin	Moxifloxacin	Delamanid	Bedaquiline	Levofloxacin	Moxifloxacin	Delamanid	Bedaquiline	Levofloxacin	Moxifloxacin	Delamanid	Bedaquiline
Cost, million USD	46.1 (26.5 - 77.7)	102 (75.1 - 140)	104 (78.8 - 142)	106 (80 - 144)	114 (88.3 - 151)	106 (80.4 - 143)	112 (86.5 - 148)	115 (89.4 - 151)	138 (110 - 174)	115 (89.2 - 148)	119 (93.5 - 154)	124 (97.8 - 159)	161 (129 - 199)	124 (96.7 - 158)
Deaths	5,220 (4,460 - 6,100)	3,210 (2,670 - 3,810)	2,460 (2,060 - 2,890)	2,460 (2,060 - 2,890)	2,260 (1,890 - 2,670)	2,260 (1,890 - 2,670)	2,210 (1,860 - 2,610)	2,210 (1,860 - 2,610)	1,940 (1,630 - 2,280)	1,940 (1,630 - 2,280)	2,140 (1,800 - 2,520)	2,140 (1,800 - 2,520)	1,850 (1,550 - 2,170)	1,850 (1,550 - 2,170)
Life-years lost, 3% discount	146,000 (124,000 - 171,000)	89,500 (74,300 - 107,000)	68,600 (57,200 - 81,000)	68,600 (57,200 - 81,000)	62,900 (52,400 - 74,700)	62,900 (52,400 - 74,700)	61,700 (51,800 - 73,000)	61,700 (51,800 - 73,000)	54,100 (45,300 - 63,800)	54,100 (45,300 - 63,800)	59,700 (50,000 - 70,500)	59,700 (50,000 - 70,500)	51,500 (43,100 - 60,800)	51,500 (43,100 - 60,800)
Incremental cost, million USD	0	55.6 (35.1 - 83.1)	58.3 (37.5 - 86.4)	59.5 (38.7 - 87.9)	67.6 (46.4 - 96.7)	59.6 (38.9 - 88.2)	66 (44 - 94.7)	68.9 (46.9 - 97.9)	92 (66.9 - 121)	68.6 (46 - 96.2)	73.1 (49.9 - 102)	77.5 (54.1 - 107)	115 (85.2 - 146)	77.6 (52.9 - 108)
Incremental deaths	0	-2,010 (-2,400 - -1,660)	-2,760 (-3,270 - -2,290)	-2,760 (-3,270 - -2,290)	-2,960 (-3,510 - -2,470)	-2,960 (-3,510 - -2,470)	-3,010 (-3,550 - -2,520)	-3,010 (-3,550 - -2,520)	-3,280 (-3,860 - -2,760)	-3,280 (-3,860 - -2,760)	-3,080 (-3,640 - -2,590)	-3,080 (-3,640 - -2,590)	-3,380 (-3,970 - -2,840)	-3,380 (-3,970 - -2,840)
Incremental life-years saved, 3% discount	0	56,300 (46,300 - 67,500)	77,200 (63,700 - 91,800)	77,200 (63,700 - 91,800)	82,900 (68,700 - 98,300)	82,900 (68,700 - 98,300)	84,100 (70,100 - 99,400)	84,100 (70,100 - 99,400)	91,800 (76,900 - 108,000)	91,800 (76,900 - 108,000)	86,100 (72,100 - 102,000)	86,100 (72,100 - 102,000)	94,300 (79,100 - 112,000)	94,300 (79,100 - 112,000)
ICER, USD/DALY	-	989	755	771	816	719	785	820	1003	748	849	900	1216	823

Sensitivity analysis around discount rate

Table 8 Sensitivity additionally using 1% and 5% discount rates

discount rate	ICER, $/DALY	target group	TPT regimen
3%	**960**	HHCM, no PT	none
1%	547	HHCM, no PT	none
5%	1461	HHCM, no PT	none
3%	**738**	PT to <5/HIV+	Lfx
1%	420	PT to <5/HIV+	Lfx
5%	1124	PT to <5/HIV+	Lfx
3%	**754**	PT to <5/HIV+	MXF
1%	429	PT to <5/HIV+	MXF
5%	1148	PT to <5/HIV+	MXF
3%	**799**	PT to <5/HIV+	DLM
1%	454	PT to <5/HIV+	DLM
5%	1218	PT to <5/HIV+	DLM
3%	**703**	PT to <5/HIV+	BDQ
1%	400	PT to <5/HIV+	BDQ
5%	1072	PT to <5/HIV+	BDQ
3%	**773**	PT to <5/HIV+/TST+	Lfx
1%	440	PT to <5/HIV+/TST+	Lfx
5%	1176	PT to <5/HIV+/TST+	Lfx
3%	**807**	PT to <5/HIV+/TST+	MXF
1%	460	PT to <5/HIV+/TST+	MXF
5%	1229	PT to <5/HIV+/TST+	MXF
3%	**992**	PT to <5/HIV+/TST+	DLM
1%	565	PT to <5/HIV+/TST+	DLM
5%	1510	PT to <5/HIV+/TST+	DLM
3%	**737**	PT to <5/HIV+/TST+	BDQ
1%	420	PT to <5/HIV+/TST+	BDQ
5%	1122	PT to <5/HIV+/TST+	BDQ
3%	**838**	PT to <15	Lfx
1%	478	PT to <15	Lfx
5%	1275	PT to <15	Lfx
3%	**890**	PT to <15	MXF
1%	507	PT to <15	MXF
5%	1354	PT to <15	MXF
3%	**1208**	PT to <15	DLM
1%	688	PT to <15	DLM
5%	1838	PT to <15	DLM
3%	**814**	PT to <15	BDQ
1%	464	PT to <15	BDQ
5%	1238	PT to <15	BDQ

In Table 8 we show global ICERs for 1% and 5% discount rates (as well as our main analysis value of 3%, shown in bold) for each regimen and target group. This is to enable assessment of the impact of higher and lower discounting of future life-years. Discounting future life-years for children more strongly can increase the ICER (by a mean factor of 1.52), whereas discounting less decreases the ICER (by a mean factor of 0.57). Revised versions of Figure 3 are shown in Figure 12 (using 1% discount rate) and Figure 13 (using 5% discount rate). In the GitHub repository are versions of Figure 10 for 1% and 5% discount rates, and versions of Figure 11 for 1% and 5% discount rates.

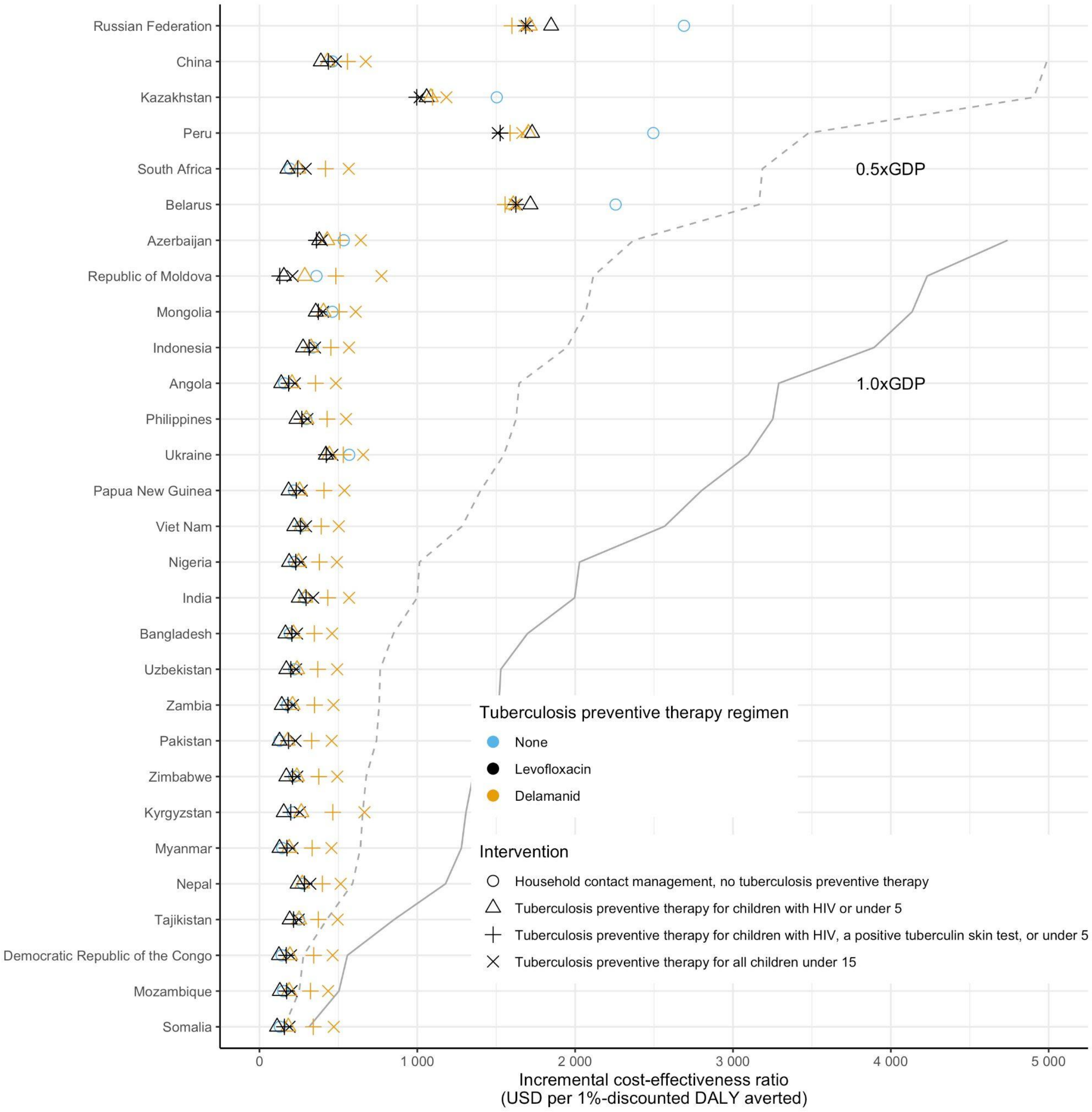

Figure 12 Figure 3 in article revised with a discount rate of 1%

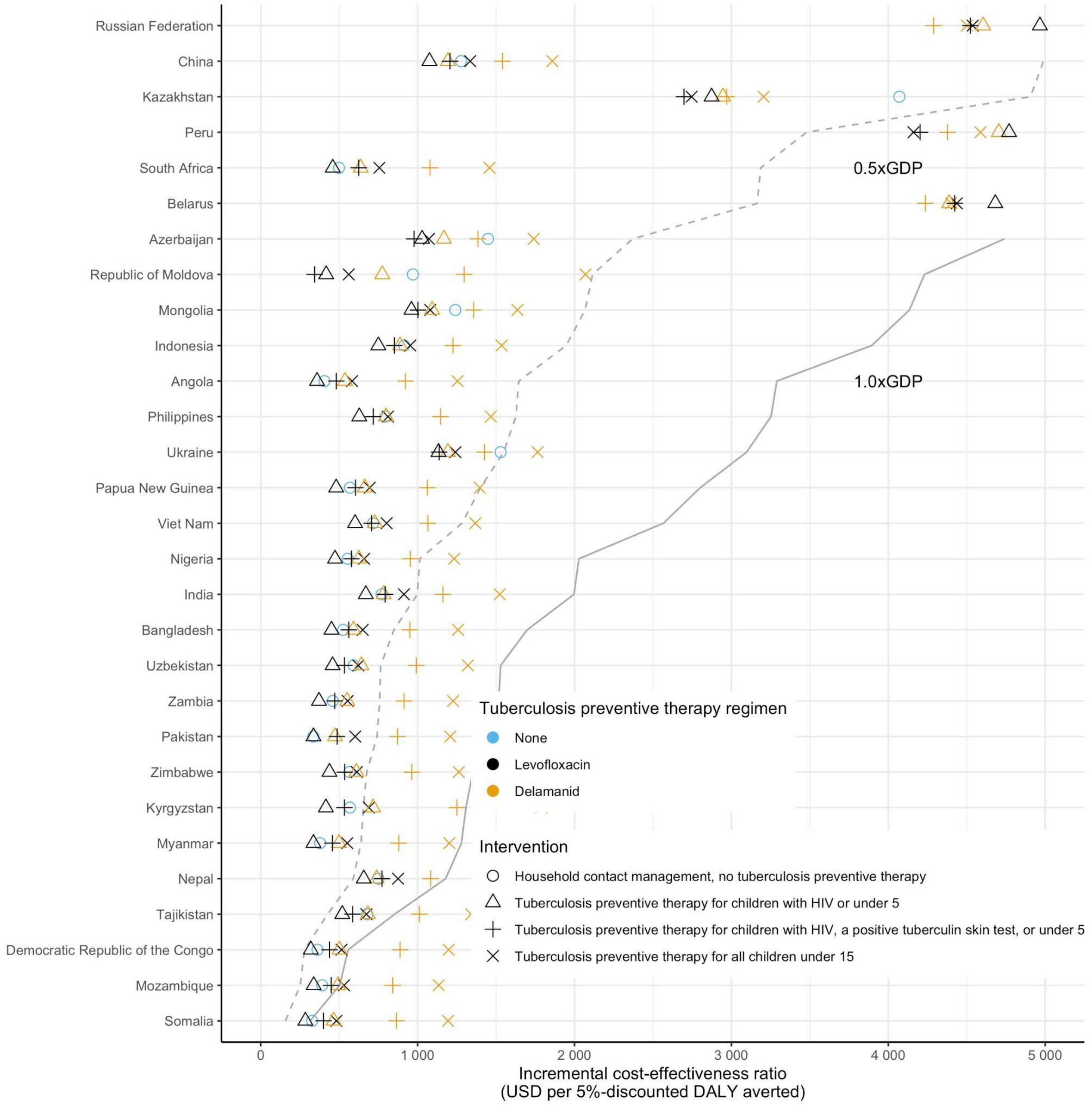

Figure 13 Figure 3 in article revised with a discount rate of 5%

Adverse event estimates

Table 9 reports estimates of adverse events and serious adverse events by WHO region and age group, showing 95% uncertainty intervals. We did not vary our assumptions on adverse event rates by regimen.

Table 9 Estimates of adverse and serious adverse events by WHO region and age group

WHO region	Age cateogory (years)	Adverse Events	Serious Adverse Events
**AMR**	[0,5)	354 (236 - 510)	73.7 (29.1 - 142)
**AMR**	[5,15)	392 (261 - 560)	81.6 (32 - 157)
**EMR**	[0,5)	681 (458 - 963)	142 (55.7 - 268)
**EMR**	[5,15)	720 (471 - 1,010)	150 (58.2 - 280)
**AFR**	[0,5)	3,070 (1,980 - 4,420)	640 (246 - 1,250)
**AFR**	[5,15)	3,290 (2,140 - 4,720)	686 (265 - 1,330)
**EUR**	[0,5)	3,120 (2,100 - 4,310)	650 (260 - 1,270)
**EUR**	[5,15)	2,820 (1,900 - 3,860)	588 (232 - 1,120)
**WPR**	[0,5)	1,810 (764 - 3,750)	376 (101 - 908)
**WPR**	[5,15)	1,870 (773 - 3,880)	390 (100 - 957)
**SEA**	[0,5)	7,770 (5,280 - 10,700)	1,620 (614 - 3,210)
**SEA**	[5,15)	7,930 (5,340 - 11,100)	1,660 (627 - 3,220)

List of countries included

List of ISO3 codes for the 213 countries included in out results:

ABW, AFG, AGO, AIA, ALB, AND, ARE, ARG, ARM, ASM, ATG, AUS, AUT, AZE, BDI, BEL, BEN, BFA, BGD, BGR, BHR, BHS, BIH, BLR, BLZ, BMU, BOL, BRA, BRB, BRN, BTN, BWA, CAF, CAN, CHE, CHL, CHN, CIV, CMR, COD, COG, COK, COL, COM, CPV, CRI, CUB, CUW, CYM, CYP, CZE, DEU, DJI, DMA, DNK, DOM, DZA, ECU, EGY, ERI, ESP, EST, ETH, FIN, FJI, FRA, GAB, GBR, GEO, GHA, GIN, GMB, GNB, GNQ, GRC, GRD, GRL, GTM, GUM, GUY, HKG, HND, HRV, HTI, HUN, IDN, IND, IRL, IRN, IRQ, ISL, ISR, ITA, JAM, JOR, JPN, KAZ, KEN, KGZ, KHM, KIR, KNA, KOR, KWT, LAO, LBN, LBR, LBY, LCA, LKA, LSO, LTU, LUX, LVA, MAC, MAR, MCO, MDA, MDG, MDV, MEX, MHL, MKD, MLI, MLT, MMR, MNE, MNG, MNP, MOZ, MRT, MSR, MUS, MWI, MYS, NAM, NCL, NER, NGA, NIC, NIU, NLD, NOR, NPL, NRU, NZL, OMN, PAK, PAN, PER, PHL, PLW, PNG, POL, PRI, PRK, PRT, PRY, PYF, QAT, ROU, RUS, RWA, SAU, SDN, SEN, SGP, SLB, SLE, SLV, SMR, SOM, SRB, SSD, STP, SUR, SVK, SVN, SWE, SWZ, SXM, SYC, SYR, TCA, TCD, TGO, THA, TJK, TKL, TKM, TLS, TON, TTO, TUN, TUR, TUV, TZA, UGA, UKR, URY, USA, UZB, VCT, VEN, VGB, VNM, VUT, WLF, WSM, YEM, ZAF, ZMB, ZWE

References

1 Dodd PJ, Yuen CM, Becerra MC, Revill P, Jenkins HE, Seddon JA. Potential effect of household contact management on childhood tuberculosis: a mathematical modelling study. *The Lancet Global Health* 2018; **6**: e1329–38.

2 Martinez L, Shen Y, Handel A, *et al.* Effectiveness of WHO's pragmatic screening algorithm for child contacts of tuberculosis cases in resource-constrained settings: a prospective cohort study in Uganda. *Lancet Respir Med* 2017; published online Dec 19. DOI:10.1016/S2213-2600(17)30497-6.

3 Sayedi SM, Seddiq MK, Rashidi MK, *et al.* Active household contact screening for tuberculosis and provision of isoniazid preventive therapy to under-five children in Afghanistan. *PLoS One* 2020; **15**: e0240031.

4 Dodd PJ, Sismanidis C, Seddon JA. Global burden of drug-resistant tuberculosis in children: a mathematical modelling study. *Lancet Infect Dis* 2016; **16**: 1193–201.

5 Fox GJ, Barry SE, Britton WJ, Marks GB. Contact investigation for tuberculosis: a systematic review and meta-analysis. *Eur Respir J* 2013; **41**: 140–56.

6 Shah NS, Yuen CM, Heo M, Tolman AW, Becerra MC. Yield of contact investigations in households of patients with drug-resistant tuberculosis: systematic review and meta-analysis. *Clin Infect Dis* 2014; **58**: 381–91.

7 Martinez L, Cords O, Robert Horsburgh C, *et al.* The risk of tuberculosis in children after close exposure: a systematic review and individual-participant meta-analysis. *Lancet* 2020; **395**: 973–84.

8 Dodd PJ, Prendergast AJ, Beecroft C, Kampmann B, Seddon JA. The impact of HIV and antiretroviral therapy on TB risk in children: a systematic review and meta-analysis. *Thorax* 2017; **72**: 559–75.

9 Chiang SS, Brooks MB, Jenkins HE, *et al.* Concordance of drug resistance profiles between persons with drug-resistant tuberculosis and their household contacts: a systematic review and meta-analysis. *Clin Infect Dis* 2020; published online May 25. DOI:10.1093/cid/ciaa613.

10 Ayieko J, Abuogi L, Simchowitz B, Bukusi EA, Smith AH, Reingold A. Efficacy of isoniazid prophylactic therapy in prevention of tuberculosis in children: a meta-analysis. *BMC Infect Dis* 2014; **14**: 91.

11 Smieja MJ, Marchetti CA, Cook DJ, Smaill FM. Isoniazid for preventing tuberculosis in non-HIV infected persons. *Cochrane Database Syst Rev* 2000; : CD001363.

12 Zunza M, Gray DM, Young T, Cotton M, Zar HJ. Isoniazid for preventing tuberculosis in HIV-infected children. *Cochrane Database Syst Rev* 2017; **8**: CD006418.

13 Seddon JA, Hesseling AC, Finlayson H, *et al.* Preventive therapy for child contacts of multidrug-resistant tuberculosis: a prospective cohort study. *Clin Infect Dis* 2013; **57**: 1676–84.

14 Malik AA, Becerra MC, Lash TL, *et al.* Risk Factors for Adverse Events in Household Contacts Prescribed Preventive Treatment for Drug-resistant Tuberculosis Exposure. *Clin Infect Dis* 2021; **72**: 1709–15.

15 Jenkins HE, Yuen CM, Rodriguez CA, *et al.* Mortality in children diagnosed with tuberculosis: a systematic review and meta-analysis. *Lancet Infect Dis* 2017; **17**: 285–95.

16 Dodd PJ, Yuen CM, Sismanidis C, Seddon JA, Jenkins HE. The global burden of tuberculosis mortality in children: a mathematical modelling study. *Lancet Glob Health* 2017; **5**: e898–906.

17 Harausz EP, Garcia-Prats AJ, Law S, *et al.* Treatment and outcomes in children with multidrug-resistant tuberculosis: A systematic review and individual patient data meta-analysis. *PLoS Med* 2018; **15**: e1002591.

18 Dodd PJ, Yuen CM, Jayasooriya SM, van der Zalm MM, Seddon JA. Quantifying the global number of tuberculosis survivors: a modelling study. *Lancet Infect Dis* 2021; published online Feb 25. DOI:10.1016/S1473-3099(20)30919-1.

19 Jo Y, Gomes I, Flack J, *et al.* Cost-effectiveness of scaling up short course preventive therapy for tuberculosis among children across 12 countries. *EClinicalMedicine* 2021; : 100707.

20 World Health Organization. Rapid communication on updated guidance on the management of tuberculosis in children and adolescents. 2021.

21 Data provided by countries to WHO and estimates of tuberculosis burden generated by WHO for the Global Tuberculosis Report. https://www.who.int/teams/global-tuberculosis-programme/data (accessed Dec 1, 2021).

22 PPP conversion factor, GDP (LCU per international $). https://data.worldbank.org/indicator/PA.NUS.PPP (accessed Dec 1, 2021).

23 Shah L, Rojas Peña M, Mori O, *et al.* A pragmatic stepped-wedge cluster randomized trial to evaluate the effectiveness and cost-effectiveness of active case finding for household contacts within a routine tuberculosis program, San Juan de Lurigancho, Lima, Peru. *Int J Infect Dis* 2020; **100**: 95–103.

24 Johnson C, Dalal S, Baggaley R, Hogan D. Annex 5. Systematic review of HIV testing costs in high and low income settings. *[online] Available at ...* 2017. https://europepmc.org/books/n/who179870/annex5/?extid=26378328&src=med.

25 Steffen RE, Pinto M, Kritski A, Trajman A. Cost-effectiveness of newer technologies for the diagnosis of Mycobacterium tuberculosis infection in Brazilian people living with HIV. *Sci Rep* 2020; **10**: 21823.

26 Stenberg K, Lauer JA, Gkountouras G, Fitzpatrick C, Stanciole A. Econometric estimation of WHO-CHOICE country-specific costs for inpatient and outpatient health service delivery. *Cost Eff Resour Alloc* 2018; **16**: 11.

27 Paediatric Operational Sustainability Expertise Exchange group. Paediatric Operational Sustainability Expertise Exchange group (POSEE group) budgeting tools. https://www.dropbox.com/sh/rmzlqoot9m1muxe/AACOPh8SuS4WwMQD_vfN-oHCa?dl=0. (accessed Feb 18, 2021).

28 Sarin S, Huddart S, Raizada N, *et al.* Cost and operational impact of promoting upfront GeneXpert MTB/RIF test referrals for presumptive pediatric tuberculosis patients in India. *PLoS One* 2019; **14**: e0214675.

29 Tucker A, Oyuku D, Nalugwa T, *et al.* Costs along the TB diagnostic pathway in Uganda. *Int J Tuberc Lung Dis* 2021; **25**: 61–3.

30 Stop TB Partnership | Global Drug Facility. Global Drug Facility (GDF) Medicines catalogue.

2021.

31 Adam T, Evans DB, Murray CJL. Econometric estimation of country-specific hospital costs. *Cost Eff Resour Alloc* 2003; **1**: 3.

32 World Health Organization. Global tuberculosis report 2019. Geneva: World Health Organization, 2019.

33 Global Tuberculosis Reports. https://www.who.int/teams/global-tuberculosis-programme/tb-reports (accessed Dec 1, 2021).

34 GDP (current US$). https://data.worldbank.org/indicator/NY.GDP.MKTP.CD (accessed Dec 1, 2021).

35 The Global Drug Facility (GDF). https://www.stoptb.org/what-we-do/global-drug-facility-gdf (accessed Dec 1, 2021).

36 Ochalek J, Lomas J, Claxton K. Estimating health opportunity costs in low-income and middle-income countries: a novel approach and evidence from cross-country data. *BMJ Glob Health* 2018; **3**: e000964.

37 Woods B, Revill P, Sculpher M, Claxton K. Country-Level Cost-Effectiveness Thresholds: Initial Estimates and the Need for Further Research. *Value Health* 2016; **19**: 929–35.

38 Chi Y-L, Blecher M, Chalkidou K, *et al.* What next after GDP-based cost-effectiveness thresholds? *Gates Open Res* 2020; **4**: 176.
